# Technology dictates algorithms: recent developments in read alignment

**DOI:** 10.1186/s13059-021-02443-7

**Published:** 2021-08-26

**Authors:** Mohammed Alser, Jeremy Rotman, Dhrithi Deshpande, Kodi Taraszka, Huwenbo Shi, Pelin Icer Baykal, Harry Taegyun Yang, Victor Xue, Sergey Knyazev, Benjamin D. Singer, Brunilda Balliu, David Koslicki, Pavel Skums, Alex Zelikovsky, Can Alkan, Onur Mutlu, Serghei Mangul

**Affiliations:** 1 Computer Science Department, ETH Zürich, 8092 Zürich, Switzerland; 2 Computer Engineering Department, Bilkent University, 06800 Bilkent, Ankara, Turkey; 3 Information Technology and Electrical Engineering Department, ETH Zürich, Zürich 8092 Switzerland; 4 Department of Computer Science, University of California Los Angeles, Los Angeles, CA 90095 USA; 5 Department of Clinical Pharmacy, School of Pharmacy, University of Southern California, Los Angeles, CA 90089 USA; 6 Department of Epidemiology, Harvard T.H. Chan School of Public Health, Boston, MA 02115 USA; 7 Program in Medical and Population Genetics, Broad Institute of MIT and Harvard, Cambridge, MA 02142 USA; 8 Department of Computer Science, Georgia State University, Atlanta, GA 30302 USA; 9 Bioinformatics Interdepartmental Ph.D. Program, University of California Los Angeles, Los Angeles, CA 90095 USA; 10 Division of Pulmonary and Critical Care Medicine, Northwestern University Feinberg School of Medicine, Chicago, IL 60611 USA; 11 Department of Biochemistry & Molecular Genetics, Northwestern University Feinberg School of Medicine, Chicago, USA; 12 Simpson Querrey Institute for Epigenetics, Northwestern University Feinberg School of Medicine, Chicago, IL 60611 USA; 13 Department of Computational Medicine, University of California Los Angeles, Los Angeles, CA 90095 USA; 14 Computer Science and Engineering, Pennsylvania State University, University Park, PA 16801 USA; 15 Biology Department, Pennsylvania State University, University Park, PA 16801 USA; 16 The Huck Institutes of the Life Sciences, Pennsylvania State University, University Park, PA 16801 USA; 17 The Laboratory of Bioinformatics, I.M. Sechenov First Moscow State Medical University, Moscow, 119991 Russia; 18 Bilkent-Hacettepe Health Sciences and Technologies Program, Ankara, Turkey

## Abstract

Aligning sequencing reads onto a reference is an essential step of the majority of genomic analysis pipelines. Computational algorithms for read alignment have evolved in accordance with technological advances, leading to today’s diverse array of alignment methods. We provide a systematic survey of algorithmic foundations and methodologies across 107 alignment methods, for both short and long reads. We provide a rigorous experimental evaluation of 11 read aligners to demonstrate the effect of these underlying algorithms on speed and efficiency of read alignment. We discuss how general alignment algorithms have been tailored to the specific needs of various domains in biology.

**Supplementary Information:**

The online version contains supplementary material available at 10.1186/s13059-021-02443-7.

## Introduction

In April 2003, the high-throughput sequencing era started with the Human Genome Project, which led to the successful sequencing of a nearly complete human genome and establishment of a reference genome that is still in use [1]. The Human Genome Project cost approximately $3 billion over 13 years to sequence the genome of an individual human. Recent advances in high-throughput sequencing technologies have enabled cost-effective and time-efficient probing of the DNA sequences of living organisms through a process known as DNA sequencing [2]. Modern high-throughput sequencing techniques are capable of producing millions of nucleotide sequences of an individual’s DNA [3] and providing multifold coverage of whole genomes or particular genomic regions. The output of high-throughput sequencing consists of sets of relatively short genomic sequences, usually referred to as *reads*. Contemporary sequencing technologies are capable of generating tens of millions to billions of reads per sample, with read lengths ranging from a few hundred to a few million base pairs [4].

The trade-off for decreased cost and increased throughput offered by modern sequencing technologies is a larger margin of noise in sequencing data [5]. The magnitude of error rates in data produced by state-of-the-art sequencing platforms varies from ~ 10^−3^ for short reads to ~ 15 × 10^−2^ for the relatively new long and ultra-long reads [6]. The increased error rate of today’s emerging long-read technologies may negatively impact biological interpretations. For example, errors in protein-coding regions can bias the accuracy of protein predictions [7]. Sequenced reads lack information about the order and origin (i.e., which part, homolog, and strand of the subject genome) of reads. The main challenge in genome analysis today is to reconstruct the complete genome of an individual. This process, *read alignment* (also known as *read mapping*), typically requires the reference genome which is used to determine the potential location of each read. Accuracy of alignment has a strong effect on many downstream analyses [8]. For example, most trans-eQTL signals were shown to be solely caused by alignment errors [9].

Read alignment can be performed in a brute force manner but is impractical for modern sequencing platforms capable of producing hundreds of millions of reads. Instead, today’s efficient bioinformatics algorithms enable fast and accurate read alignment and can be thousands of orders of magnitude faster when compared to the naive brute force approach [10] (Supplementary Note 1). Read alignment enables observation of the differences between the read and the reference genome. These differences can be caused by either real genetic variants in the sequenced genome or errors generated by the sequencing platform. These sequencing errors and read lengths, which are typically short, make the read alignment problem computationally challenging. The continued increase in the throughput of modern sequencing technologies creates additional demand for efficient algorithms for read alignment. Over the past several decades, a plethora of tools were developed to align reads onto reference genomes across various domains of biology. Previous efforts that provide overviews of various algorithms and techniques used by read aligners are presented elsewhere [10–12], including studies that present benchmarks of existing tools [13, 14]. Since the time those efforts were published, many new alignment algorithms have been developed. Additionally, previous efforts lack a historical perspective on algorithm development.

Our review provides a historical perspective on how technological advancements in sequencing are shaping algorithm development across various domains of modern biology, and we systematically assess the underlying algorithms of a large number of aligners (*n* = 107). Algorithmic development and challenges associated with read alignment are to a large degree data- and technology-driven, and emerging highly accurate ultra-long-read sequencing techniques promise to expand the application of read alignment.

## Where do reads come from—advantages and limitations of read alignment

One can study an individual genome using sequencing data in two ways: by mapping reads to a reference genome, if it exists, or by de novo assembling the reads. The complexity of the human genome, in combination with the short length of sequenced reads, poses substantial challenges to our ability to accurately assemble personal genomes [15]. Even recently-introduced ultra-long reads [16] (up to 2 Mb) offer the limited capacity to build a de novo assembly of an individual genome with no prior knowledge about the reference genome [16]. The presence of many repetitive regions in the human genome limits our ability to assemble a personal human genome as a single sequence. Emerging long-read sequencing technologies that are capable of producing ultra-long reads [16] promise to deliver more accurate assemblies [17]. However, the relatively high error rate of data output from recently developed long-read sequencing technologies often results in inaccuracies in the assembled genomes, especially when using low sequencing coverage [18, 19].

The read alignment problem is known to be solvable in polynomial time [20], while a polynomial-time solution for genome assembly is still unknown [20–22]. Genome assembly is typically slower and more computationally intensive than read alignment [17, 23, 24] due to the presence of repeats that are much longer than the typical read length. This makes assembly impractical in studies that involve large-scale clinical cohorts of thousands of individuals. At the same time, when the reference genome is unknown, long reads are a valuable resource for assembling genomes that are far more complex than the human genome, such as the hexaploid bread wheat genome [17, 23, 25].

The availability of a large number of alignment methods that are scalable to both read length and genome size has enabled read alignment to become an essential component of high-throughput sequencing analysis (Table 1) [26]. However, read alignment also has its own fundamental challenges. First, some challenges are caused by the incompleteness of the reference genomes that have multiple assembly gaps [16]. Reads originating from these gaps often remain unmapped or are incorrectly mapped to homologous regions. Second, the presence of repetitive regions of the genome confounds current read alignment techniques, which often map reads originating from one region to match *several* other repetitive regions (such reads are known as multi-mapped reads). In such cases, most read aligners *simply* report one location randomly selected among the possible mapping locations, in turn, significantly reducing the number of detected variants [27]. Third, read alignment techniques should tolerate differences between reads and the reference genome. These differences may correspond to a single nucleotide (including deletion, insertion, and substitution of a nucleotide) or to larger structural variants [28]. Fourth, read alignment algorithms need to align reads to both forward and reverse DNA strands of the same reference genome in order to tackle the strand bias problem, defined as the difference in genotypes identified by reads that map to forward and reverse DNA strands. Strand bias is likely caused by errors introduced during library preparation and not by mapping artifacts [27, 29].

**Table 1:** Summary of algorithms and features of the examined read alignment methods. We surveyed 107 alignment tools published from 1988 to 2020 (indicated in column “Year of publication”). The table is sorted by year of publication, and then grouped according to the area(s) of application (indicated in column “Application”) within each year. In column “Indexing,” we document the algorithms used to index the genome (the first step in read alignment). In column “Global Positioning,” we document the algorithms used to determine a global position of the read in the reference genome (the second step). In column “Pairwise alignment,” we document the algorithm used to determine the similarity between the read and the corresponding region of the reference genome (the last step). SW, NW, HD, and DP stand for Smith-Waterman algorithm, Needleman-Wunsch algorithm, Hamming distance, and dynamic programming, respectively. In column “Wrapper,” we document the read alignment algorithms that are built on top of other read alignment tools. Finally, we report the maximum read length tested in the corresponding paper in column “Max. Read Length Tested in the Paper (bp).” The tested read length in each paper is not necessarily the maximum read length that each tool can handle

Aligner	URL	Year of publication	Application	Indexing	Global Positioning			Pairwise alignment	Wrapper	Max. read length tested in the paper (bp)
					Fix length seed	Spaced seed	Seed chaining			
FASTA [30]	https://fasta.bioch.virginia.edu/fasta_www2/fasta_list2.shtml	1988	DNA	Hashing	Y	N	Y	SW and NW	N	1500
BLAST [31]	https://blast.ncbi.nlm.nih.gov/Blast.cgi	1990	DNA	Hashing	Y	N	Y	Non-DP Heuristic	N	73360
Gapped BLAST [32]	https://blast.ncbi.nlm.nih.gov/Blast.cgi	1997	DNA	Hashing	Y	N	Y	SW	N	246
SSAHA [33]	https://www.sanger.ac.uk/science/tools/ssaha	2001	DNA	Hashing	Y	N	N	NW	N	500
PatternHunter [34–37]	https://www.bioinfor.com/	2002	DNA	Hashing	Y	Y	Y	Non-DP heuristic	N	500
BLAT [38]	https://genome.ucsc.edu/cgi-bin/hgBlat	2002	DNA	Hashing	Y	N	Y	Non-DP heuristic	N	500
BLASTZ [39]	https://www.bx.psu.edu/miller_lab/	2003	DNA	Hashing	Y	N	N	SW	Y	3000
C4 [40]	https://github.com/nathanweeks/exonerate	2005	DNA	Hashing	Y	N	Y	Sparse DP	N	N/A
GMAP [41]	https://github.com/juliangehring/GMAP-GSNAP	2005	DNA	Hashing	N	N	Y	NW	N	N/A
BWT-SW [42]	https://github.com/mruffalo/bwt-sw	2008	DNA	BWT	Y	N	N	SW	N	2000
MAQ [43]	http://maq.sourceforge.net/maq-man.shtml	2008	DNA	Hashing	Y	Y	N	SW	N	63
RMAP [44]	https://github.com/smithlabcode/rmap	2008	DNA	Hashing	Y	N	N	HD	N	36
SOAP [45]	https://github.com/ShujiaHuang/SOAPaligner	2008	DNA	Hashing	Y	N	N	Non-DP heuristic	N	50
SOCS [46]	http://socs.biology.gatech.edu/	2008	DNA	Hashing	Y	N	N	Rabin-Karp Algorithm	N	35
SeqMap [47]	http://www-personal.umich.edu/~jianghui/seqmap/	2008	DNA	Hashing	Y	N	N	Non-DP Heuristic	N	30
ZOOM [48]	http://www.bioinfor.com/zoom-1-3-gui-release-next-gen-seq/	2008	DNA	Hashing	Y	Y	N	SW	N	36
QPALMA [49, 50]	http://www.raetschlab.org/suppl/qpalma	2008	RNA-Seq	Suffix array	Y	N	Y	SW	Y	36
BRAT [51]	http://compbio.cs.ucr.edu/brat/	2009	BS-Seq	Hashing	Y	N	N	HD	N	26
BSMAP [52]	https://github.com/genome-vendor/bsmap	2009	BS-Seq	Hashing	Y	N	N	HD	N	32
BFAST [53]	https://github.com/nh13/BFAST/	2009	DNA	Hashing	N	Y	N	SW	N	55
BWA [54]	https://github.com/lh3/bwa	2009	DNA	BWT-FM	N	N	N	Semi-Global	N	125
Bowtie [55]	http://bowtie-bio.sourceforge.net/manual.shtml	2009	DNA	BWT-FM	Y	N	N	HD	N	76
CloudBurst [56]	https://sourceforge.net/projects/cloudburst-bio/	2009	DNA	Hashing	Y	N	N	Landau-Vishkin	N	36
GNUMAP [57]	https://github.com/byucsl/gnumap	2009	DNA	Hashing	Y	N	Y	NW	N	36
GenomeMapper [58]	http://1001genomes.org/software/genomemapper_singleref.html	2009	DNA	Hashing	Y	N	Y	NW	N	200
MOM [59]	https://github.com/hugheaves/MOM	2009	DNA	Hashing	Y	N	N	HD	N	40
PASS [60]	http://pass.cribi.unipd.it/cgi-bin/pass.pl	2009	DNA	Hashing	Y	N	Y	NW	N	32
PerM [61]	https://code.google.com/archive/p/perm/downloads	2009	DNA	Hashing	Y	Y	N	HD	N	47
RazerS [62]	https://github.com/seqan/seqan/tree/master/apps/razers	2009	DNA	Hashing	Y	Y	Y	Myers Bit Vector	N	76
SHRiMP [63]	http://compbio.cs.toronto.edu/shrimp/	2009	DNA	Hashing	N	N	N	SW	N	35
SOAP2 [64]	https://github.com/ShujiaHuang/SOAPaligner	2009	DNA	BWT-FM	Y	N	N	SW	N	44
Slider [65]	http://www.bcgsc.ca/platform/bioinfo/software/slider	2009	DNA	Hashing	Y	N	N	HD	N	36
segemehl [66]	https://www.bioinf.uni-leipzig.de/Software/segemehl/	2009	DNA	Suffix array	N	N	Y	SW	N	35
TopHat [67]	https://ccb.jhu.edu/software/tophat/index.shtml	2009	RNA-Seq	BWT-FM	Y	N	N	HD	Y	42
BS-Seeker [68]	http://pellegrini-legacy.mcdb.ucla.edu/bs_seeker/BS_Seeker.html	2010	BS-Seq	BWT-FM	Y	N	N	HD	Y	36
BWA-SW [54]	https://github.com/lh3/bwa	2010	DNA	BWT-FM	N	N	N	SW	N	10000
GASSST [35]	http://www.irisa.fr/symbiose/projects/gassst/	2010	DNA	Hashing	Y	Y	Y	Semi-Global	N	500
GSNAP [37]	https://github.com/juliangehring/GMAP-GSNAP	2010	DNA	Hashing	Y	N	Y	Non-DP Heuristic	N	100
SMALT [69]	https://github.com/rcallahan/smalt	2010	DNA	Hashing	Y	N	Y	SW	N	150
Slider II [70]	http://www.bcgsc.ca/platform/bioinfo/software/SliderII	2010	DNA	Hashing	Y	N	N	HD	Y	42
VMATCH [71]	http://www.vmatch.de/	2010	DNA	Suffix array	Y	N	Y	SW	Y	N/A
mrsFAST [72]	https://github.com/sfu-compbio/mrsfast	2010	DNA	Hashing	Y	N	N	HD	N	100
MapSplice [73]	https://github.com/LiuBioinfo/MapSplice	2010	RNA-Seq	BWT-FM	Y	N	N	HD	Y	100
MicroRazerS [74]	https://github.com/seqan/seqan/tree/master/apps/micro_razers	2010	RNA-Seq	Hashing	Y	N	Y	HD	N	36
SpliceMap [75]	http://web.stanford.edu/group/wonglab/SpliceMap/	2010	RNA-Seq	Hashing	Y	N	N	HD	Y	50
Supersplat [76]	http://mocklerlab.org/tools/1/manual	2010	RNA-Seq	Hashing	N	N	N	NA	N	36
Bismark [77]	https://github.com/FelixKrueger/Bismark	2011	BS-Seq	BWT-FM	Y	N	Y	SW & NW	Y	50
LAST [78]	http://last.cbrc.jp/	2011	DNA/BS-Seq/RNA	Suffix array	N	Y	N	SW & NW	N	105
DynMap [79]	https://dl.acm.org/citation.cfm?id=2147845&dl=ACM&coll=DL	2011	DNA	Hashing	Y	N	N	NW	N	52
SHRiMP2 [80]	http://compbio.cs.toronto.edu/shrimp/	2011	DNA	Hashing	Y	Y	Y	SW	N	75
SNAP [81]	http://snap.cs.berkeley.edu/	2011	DNA	Hashing	Y	N	N	NW	N	10000
Stampy [82]	https://www.well.ox.ac.uk/project-stampy	2011	DNA	Hashing	Y	N	N	NW	N	4500
TMAP	https://github.com/iontorrent/TS/tree/master/Analysis/TMAP	2011	DNA	BWT-FM	N	N	Y	SW	N	N/A
X-Mate [83]	http://grimmond.imb.uq.edu.au/X-MATE/	2011	DNA	Hashing	N	N	N	Non-DP Heuristic	N	50
SOAPSplice [84]	http://soap.genomics.org.cn/soapsplice.html	2011	RNA-Seq	BWT-FM	Y	N	N	Non-DP Heuristic	N	150
BRAT-BW [51]	http://compbio.cs.ucr.edu/brat/	2012	BS-Seq	BWT-FM	N	N	N	HD	N	62
BLASR [85]	https://github.com/mchaisso/blasr/	2012	DNA	Suffix array	Y	N	Y	NW	N	8000
Batmis [86]	https://code.google.com/archive/p/batmis/	2012	DNA	BWT-ST	Y	N	N	HD	N	100
Bowtie2 [87]	http://bowtie-bio.sourceforge.net/bowtie2	2012	DNA	BWT-FM	Y	N	Y	SW & NW	N	400
GEM [88]	https://github.com/smarco/gem3-mapper	2012	DNA	BWT-FM	N	N	Y	SW & NW	N	150
RazerS3 [89]	https://github.com/seqan/seqan/tree/master/apps/razers3	2012	DNA	Hashing	Y	Y	Y	Banded Myers Bit Vector	N	800
SeqAlto [90]	https://web.stanford.edu/group/wonglab/seqalto/	2012	DNA	Hashing	Y	N	N	NW	N	200
SplazerS [91]	https://github.com/seqan/seqan/blob/master/apps/splazers/README	2012	DNA	Hashing	Y	N	Y	Banded Myers Bit Vector	N	150
WHAM [92]	http://pages.cs.wisc.edu/~jignesh/wham/	2012	DNA	Hashing	Y	N	N	NW	N	74
YAHA [93]	https://github.com/GregoryFaust/yaha	2012	DNA	Hashing	Y	N	Y	SW	N	10000
OSA [94]	http://www.arrayserver.com/wiki/index.php?title=OSA	2012	RNA-Seq	Hashing	Y	N	N	NA	N	100
Passion [95]	https://trac.nbic.nl/passion/	2012	RNA-Seq	Hashing	Y	N	Y	SW	Y	75
BS-Seeker2 [96]	https://github.com/BSSeeker/BSseeker2	2013	BS-Seq	BWT-FM	Y	N	Y	SW & NW	Y	250
Subread [97]	http://subread.sourceforge.net/	2013	DNA/RNA-Seq	Hashing	Y	Y	Y	SW	N	202
BWA-MEM [98]	https://github.com/lh3/bwa	2013	DNA	BWT-FM	N	N	Y	SW & NW	N	650
Masai [99]	http://www.seqan.de/projects/masai	2013	DNA	Suffix tree	N	N	Y	Banded Myers Bit Vector	N	150
NextGenMap [100]	http://cibiv.github.io/NextGenMap/	2013	DNA	Hashing	Y	N	N	SW & NW	N	250
SRmapper [101]	http://www.umsl.edu/~wongch/software.html	2013	DNA	Hashing	Y	N	N	HD	N	100
mrFAST [102]	https://github.com/BilkentCompGen/mrfast	2013	DNA	Hashing	Y	N	N	Semi-Global	N	180
CRAC [103]	http://crac.gforge.inria.fr/	2013	RNA-Seq	BWT-FM	Y	N	N	Non-DP Heuristic	N	200
STAR [104]	https://github.com/alexdobin/STAR	2013	RNA-Seq	Suffix array	N	N	Y	SW	N	5000
TopHat2 [105]	https://ccb.jhu.edu/software/tophat/index.shtml	2013	RNA-Seq	BWT-FM	Y	N	Y	SW & NW	Y	101
Subjunc [106]	http://subread.sourceforge.net/	2013	RNA-seq	Hashing	Y	Y	Y	NW	N	202
BWA-PSSM [107]	http://bwa-pssm.binf.ku.dk/	2014	DNA	BWT-FM	Y	N	N	SW	Y	100
CUSHAW3 [108]	http://cushaw3.sourceforge.net/homepage.htm#latest	2014	DNA	BWT-FM	Y	N	Y	SW & Semi-Global	N	100
Hobbes2 [109]	https://hobbes.ics.uci.edu/download.shtml	2014	DNA	Hashing	Y	N	Y	Banded Myers Bit Vector	N	100
MOSAIK [110]	https://github.com/wanpinglee/MOSAIK	2014	DNA	Hashing	Y	N	N	SW	N	100
hpg-Aligner [111]	https://github.com/opencb/hpg-aligner	2014	DNA	Suffix array	N	N	Y	SW	N	5000
mrsFAST-Ultra [112]	https://github.com/sfu-compbio/mrsfast	2014	DNA	Hashing	Y	N	N	HD	N	100
JAGuaR [113]	http://www.bcgsc.ca/platform/bioinfo/software/jaguar	2014	RNA-Seq	BWT-FM	Y	N	N	SW	Y	100
ContextMap 2 [114]	http://www.bio.ifi.lmu.de/ContextMap	2015	RNA-Seq	BWT-FM	Y	N	Y	SW & NW	Y	76
HISAT [115]	http://www.ccb.jhu.edu/software/hisat/index.shtml	2015	RNA-Seq	BWT-FM	Y	N	N	Non-DP Heuristic	N	100
ERNE 2 [116]	http://erne.sourceforge.net/	2016	DNA/BS-Seq	BWT-FM + hashing	Y	N	N	HD	N	100
GraphMap [117]	https://github.com/isovic/graphmap	2016	DNA	Hashing	Y	Y	Y	Semi-global	N	9000
NanoBLASTer [118]	https://github.com/ruhulsbu/NanoBLASTer	2016	DNA	Hashing	Y	N	Y	NW	N	7040
minimap [119]	https://github.com/lh3/minimap	2016	DNA	Hashing	Y	N	N	N/A	N	13000
rHAT [120]	https://github.com/dfguan/rHAT	2016	DNA	Hashing	Y	N	Y	SW	N	8000
KART [121]	https://github.com/hsinnan75/KART	2017	DNA	BWT-FM	N	N	Y	NW	N	7118
LAMSA [122]	https://github.com/hitbc/LAMSA	2017	DNA	BWT-FM + hashing	Y	N	Y	Sparse DP	Y	100000
DART [123]	https://github.com/hsinnan75/DART	2017	RNA-Seq	BWT-FM	N	N	Y	NW	N	251
minimap2 [124]	https://github.com/lh3/minimap2	2018	DNA/RNA-Seq	Hashing	Y	N	Y	NW	N	11628
DREAM-Yara [125]	https://gitlab.com/pirovc/dream_yara/	2018	DNA	BWT-FM	Y	N	N	Banded Myers Bit Vector	Y	150
MUMmer4 [126]	https://github.com/mummer4/mummer	2018	DNA	Suffix array	Y	N	Y	SW	Y	7821
NGMLR [127]	https://github.com/philres/ngmlr	2018	DNA	Hashing	Y	N	Y	SW	N	50000
lordFAST [128]	https://github.com/vpc-ccg/lordfast	2018	DNA	BWT-FM + hashing	N	N	Y	SW & NW	N	35489
BatMeth2 [129]	https://github.com/GuoliangLi-HZAU/BatMeth2/	2019	BS-Seq	BWT-FM	Y	N	Y	SW & NW	N	125
GraphMap2 [130]	https://github.com/lbcb-sci/graphmap2	2019	DNA/RNA-Seq	Hashing	Y	Y	Y	Semi-global	N	9000
Magic-BLAST [131]	https://github.com/ncbi/magicblast	2019	DNA/RNA-Seq	Hashing	Y	N	N	Non-DP Heuristic	N	90000
BWA-MEM2 [132]	https://github.com/bwa-mem2/bwa-mem2	2019	DNA	BWT-FM	N	N	Y	SW	N	650
HISAT2 [133]	https://ccb.jhu.edu/software/hisat2/index.shtml	2019	DNA	BWT-FM	Y	N	N	Non-DP Heuristic	N	100
deSALT [134]	https://github.com/hitbc/deSALT	2019	RNA-seq	Hashing	Y	N	Y	SW	N	8000
conLSH [135]	https://www.dropbox.com/s/3jcu4i240kyu2tc/source%20code%20conLSH_bio.tar.gz?dl=0	2020	DNA	Hashing	Y	N	Y	Sparse DP	N	8000

## Co-evolution of read alignment algorithms and sequencing technologies

Over the past few decades, we have observed an increase in the number of alignment tools developed to accommodate rapid changes in sequencing technology (Table 1). Published alignment tools use a variety of algorithms to improve the accuracy and speed of read alignment (Table 2). At the same time, the development of read alignment algorithms is impacted by rapid changes in sequencing technologies, such as read length, throughput, and error rates (Supplementary Table 1). For example, some of the first alignment algorithms (e.g., BLAT [38]) were designed to align expressed sequence tag (EST) sequences, which are 200 to 500 bp in length. Another early alignment algorithm, BLASTZ [39], was designed to align 1 Mb human contigs onto the mouse genome. After short reads became available, the majority of the algorithms have focused on the problem of aligning hundreds of millions of short reads to a reference genome. Recent sequencing technologies are capable of producing multi-megabase reads at the cost of high error rates (up to 20%)—a development that poses additional challenges for modern read alignment methods [17]. A recent improvement in circular consensus sequencing (CCS) allows a substantial reduction in sequencing error rates; for example, the error rate has dropped from 15% down to 0.0001% by sequencing the same molecule at least 30 times and further correcting errors by calculating consensus [136].

**Table 2:** Advantages and limitations of read alignment algorithms. We compare the ease of implementing each algorithm (“Easy to implement”). We define the “ease of implementation” as the ability to quickly implement such an algorithm and its indexing technique, flexibly apply some changes to it, and easily understand its working principle. We also record whether the algorithm allows for an exact and/or inexact match (“Search for exact/inexact match”). The use of spaced seeds enables searching for inexact match using a hash table. We also compare the size of the genome index (indicated in column “Index size”), the speed of seed query (indicated in column “Seed query speed”), and the possibility to vary the length of the seed (“Seed length”)

	Hashing	Suffix tree and BWT-FM
Easy to implement	Yes	No
Search for exact/inexact match	Exact	Exact and inexact
Index size	Large	Compressed (small)
Indexing time	Small	Large
Seed query speed	O(1), fast	Slow
Seed length	Fixed length per index	Can be fixed or variable

**Fig. 1:**
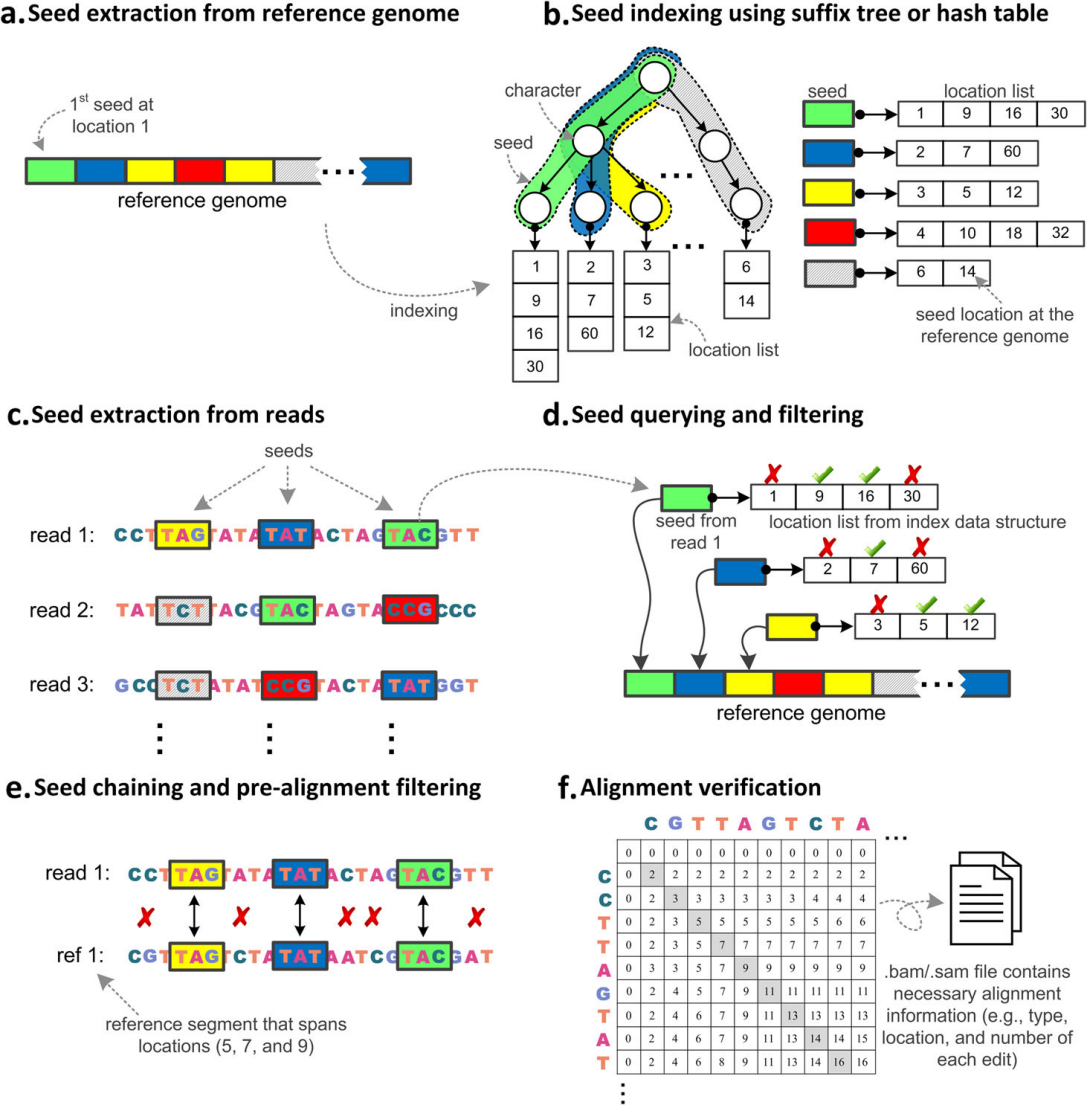
Overview of a read alignment algorithm. **a** The seeds from the reference genome sequence are extracted. **b** Each extracted seed and all its occurrence locations in the reference genome are stored using the data structure of choice (suffix tree and hash table are presented as an example). Common prefixes of the seeds are stored once in the branches of the suffix tree, while the hash table stores each seed individually. **c** The seeds from each read sequence are extracted. **d** The occurrences of each extracted seed in the reference genome are determined by querying the index database. In this example, the three seeds from the first read appear adjacent at locations 5, 7, and 9 in the reference genome. Two of the same seeds appear also adjacent at another two locations (12 and 16). Other non-adjacent locations are filtered out (marked with X) as they may not span a good match with the first read. **e** The adjacent seeds are linked together to form a longer chain of seeds by examining the mismatches between the gaps. Pre-alignment filters can also be applied to quickly decide whether or not the computationally expensive DP calculation is needed. **f** Once the pre-alignment filter accepts the alignment between a read and a region in the reference genome, then DP-based (or non-DP-based) verification algorithms are used to generate the alignment file (in BAM or SAM formats), which contains alignment information such as the exact number of differences, location of each difference, and their type.

We have studied the underlying algorithms of 107 read alignment tools that were designed for the short- and long-read sequencing technologies and were published from 1988 to 2020 (Table 1). We defined read alignment as a three-step procedure (Supplementary Note 2). First, indexing with the aim of quickly locating genomic subsequences in the reference genome is performed. This step includes building a large index database from a reference genome and/or the set of reads (Fig. 1a, b). Second, global positioning is performed to determine the potential positions of each read in the reference genome. In this step, alignment algorithms use the prepared index to determine one or more possible regions of the reference genome that are likely to be similar to each read sequence (Fig. 1c, d). Lastly, pairwise alignment is performed between the read and each of the corresponding regions of the reference genome to determine the exact number, location, and type of differences between the read and corresponding region (Fig. 1e, f).

## Hashing is the most popular technique for indexing the reference genome

The key goal of the indexing step is to facilitate quick and efficient querying over the whole reference genome sequence, producing a minimal memory footprint by storing the redundant subsequences of the reference genome only once [17, 20, 137]. Rapid advances in sequencing technologies have shaped the development of read alignment algorithms, and major changes in technology have rendered many tools obsolete. For example, some early methods [43, 44, 47, 48, 80] built the index database from the reads. Today’s longer read lengths and increased throughput of sequencing technologies make such an approach infeasible for analyzing modern sequencing data. Modern alignment algorithms typically build the index database from the reference genome and then use the subsequences of the reads (known as seeds or qgrams) to query the index database (Fig. 1a). In general, indexing the reference genome compared to the read set is a more practical and resource-frugal solution. Additionally, it allows reusing the constructed reference genome index across multiple samples.

**Fig. 2:**
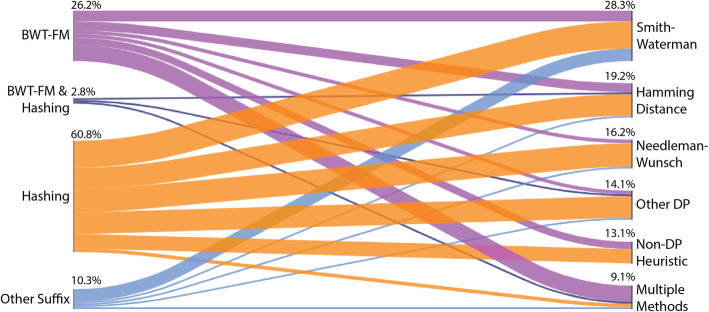
Combination of algorithms utilized by read alignment tools. Sankey plot displaying the flow of surveyed tools using each indexing technique and pairwise alignment. For every indexing technique, the percentage of surveyed tools using the algorithm is displayed (BWT-FM 26.2%, BWT-FM, and Hashing 2.8%, Hashing 60.8%, Other Suffix 10.3%). For every pairwise alignment technique, the percentage of surveyed tools using the algorithm is displayed (Smith-Waterman 28.3%, Hamming distance 19.2%, Needleman-Wunsch 16.2%, Other DP 14.1%, Non-DP Heuristic 13.1%, Multiple Methods 9.1%)

**Fig. 3:**
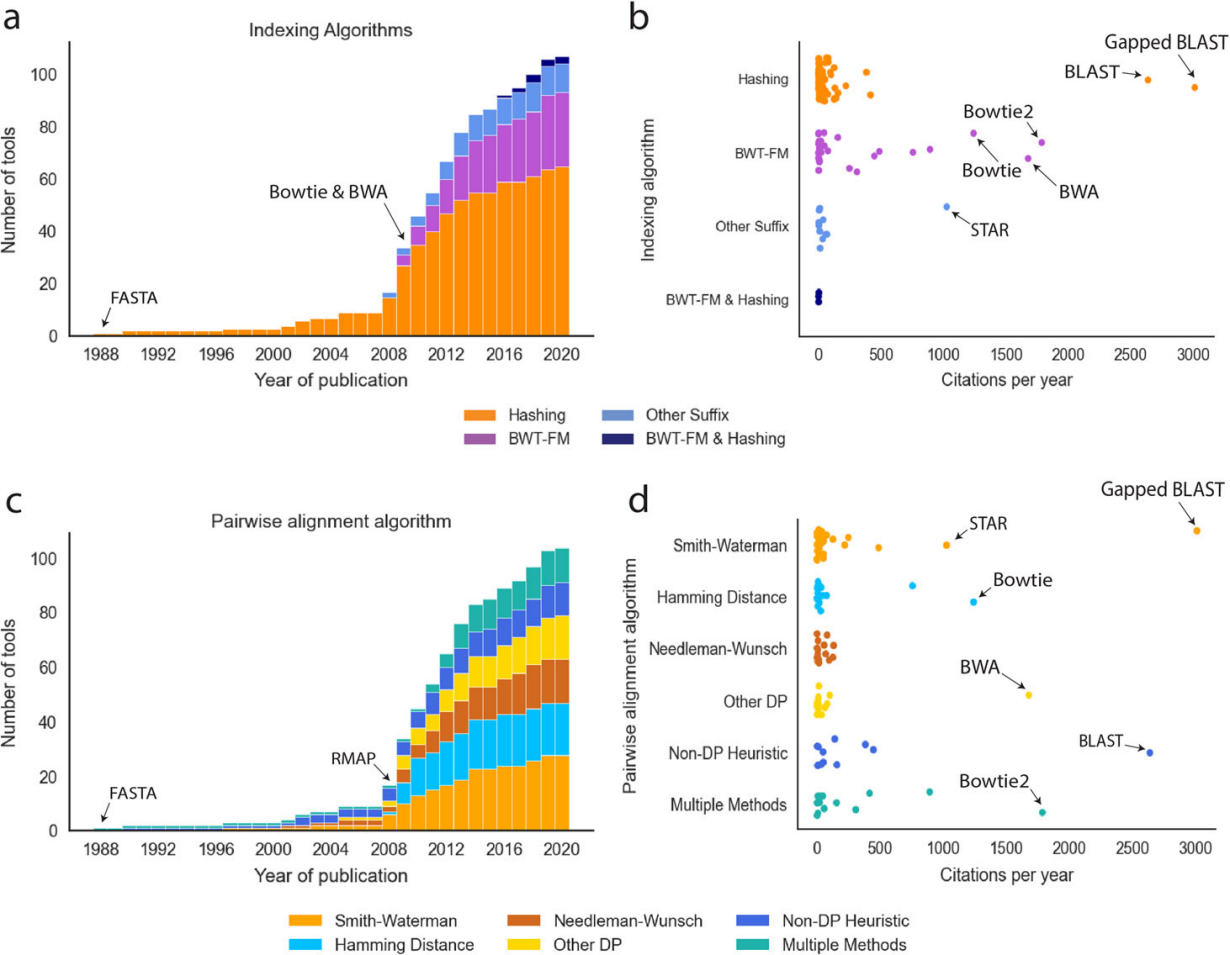
The landscape of read alignment algorithms published from 1988 to 2020. **a** Histogram showing the cumulation of surveyed tools over time colored by the algorithm used for genome indexing. The first published aligner, FASTA, is labeled as well as the point at which Bowtie and BWA were introduced and changed the landscape of aligners. **b** The popularity of all surveyed aligners, judged by citations per year since the initial release. Tools are grouped by the algorithm used for genome indexing. The six overall most popular aligners are labeled. **c** Histogram showing the cumulation of surveyed tools over time colored by the algorithm used for pairwise alignment. The two aligners credited to have been the first to use the three most popular algorithms (FASTA: Smith-Waterman and Needleman-Wunsch, RMAP: Hamming distance) are labeled. **d** The popularity of each surveyed aligner, judged by citations per year since the initial release. Tools are grouped by the algorithm used for pairwise alignment. The six overall most popular aligners are labeled.

We observe that the most popular indexing technique used by read alignment tools is hashing, which is used exclusively by 60.8% of our surveyed read aligner tools from various domains of biological research (Fig. 2). Hashing is also the most popular individual indexing method for aligners that can handle DNA-Seq data, accounting for 68.3% of the surveyed read aligner tools. Hash table indexing was first used in 1988 by FASTA [30, 138] and has since dominated the landscape of read alignment tools. Hashing was also the only dominant technique to be used until the BWT-FM index was introduced by Bowtie [55] (Fig. 3a). Its popularity can be explained by the simplicity and ease of implementation when compared to other indexing techniques. Other advantages and limitations of hashing are outlined in Table 2. The hash table is a data structure that stores the content of some short regions of the genome (e.g., seeds) and their corresponding locations in the reference genome (Fig. 1b). Such regions are also known as *k*-mers or qgrams [139]. After the genomic seeds are produced, the alignment algorithm extracts the seeds from each read and uses them as a key to query the hash table index. The hash table returns a location list storing all occurrence locations of the read seed in the reference genome.

## Alignment tools utilizing suffix-tree-based indexing are generally faster and more widely used

The second most popular approach to indexing is the suffix-tree-based techniques, used exclusively by 36.5% of the surveyed read aligner tools (Fig. 2) (Table 1). ERNE 2 [116], LAMSA [122], and lordFAST [128] are categorized separately since they combine hashing with a suffix-tree-based technique. A suffix tree is a tree-like data structure where separate branches represent different suffixes of the genome; the shared prefix between two suffixes of the genome is stored only once. Every leaf node of the suffix tree stores all occurrence locations of this unique suffix in the reference genome (Fig. 1b). Unlike a hash table, a suffix tree allows searching for both exact and inexact match seeds [140, 141] by walking through the tree branches from the root to a leaf node, detouring as needed, following the query sequence (Table 2). While some algorithms [142, 143] specifically rely on creating suffix trees, the most frequently chosen tools from this category use the Burrows-Wheeler Transform (BWT) and the FM index (hence called BWT-FM-based tools) to mimic the suffix-tree traversal process while generating a smaller memory footprint [99]. The performance of the read aligners in this category degrades as either the sequencing error rate increases or the genetic differences between the subject and the reference genome are more likely to occur [144, 145].

## The effect of read alignment algorithms on speed of alignment and computational resources

**Fig. 4:**
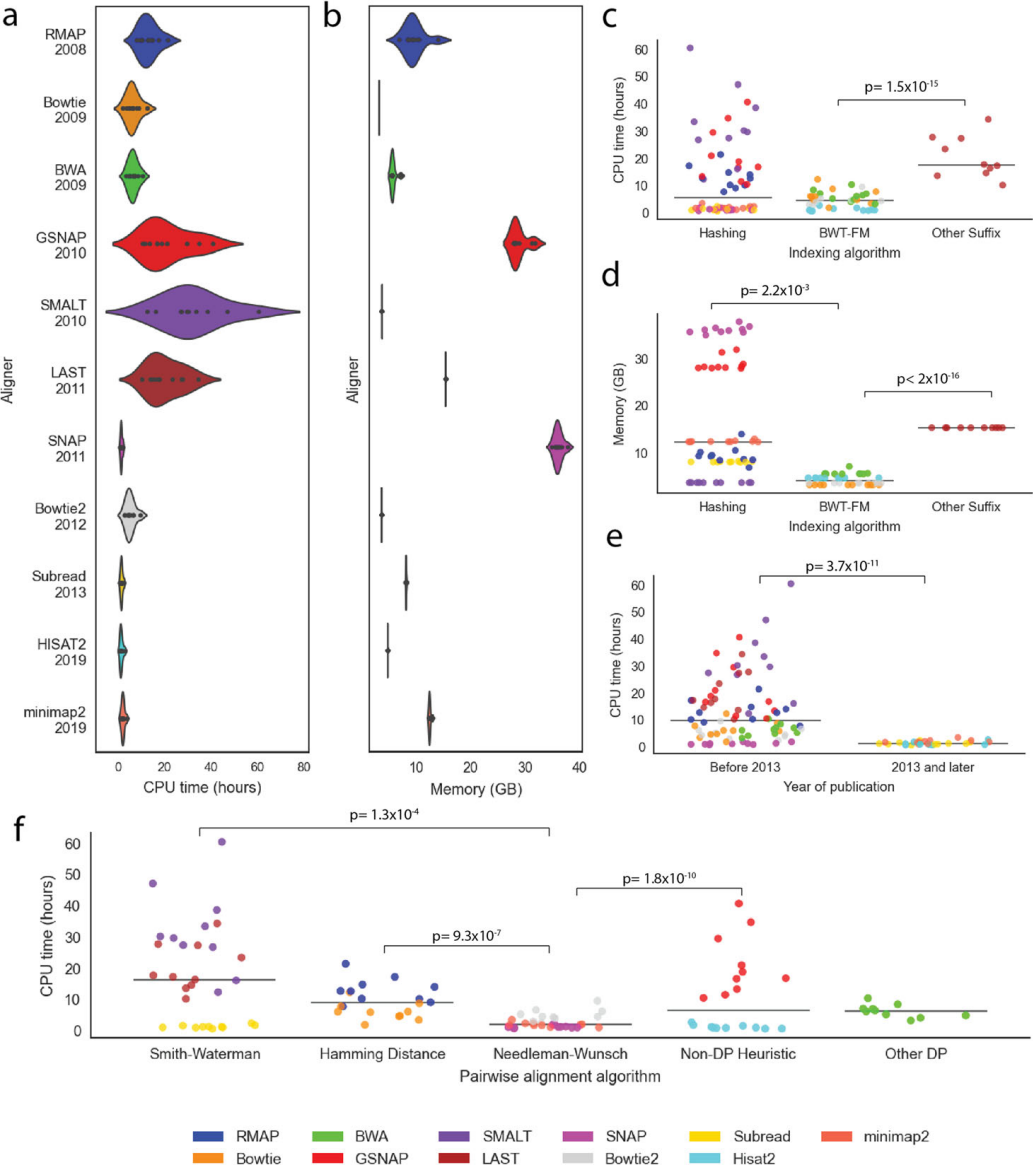
The effect of read alignment algorithms on the speed of alignment and computational resources. Results of the benchmarking performed on 11 surveyed DNA read alignment tools that can be installed through bioconda (RMAP, Bowtie, BWA, GSNAP, SMALT, LAST, SNAP, Bowtie2, Subread, HISAT2, and minimap2) additionally noted in Supplementary Table 2 and Supplementary Note 3. Each tool’s CPU time and RAM required were recorded for 10 different WGS samples from the 1000 Genomes Project. **a, b** Violin plots showing the relative performance (**a** CPU time and **b** RAM) of the benchmarked aligners. Aligners are ordered by year of release. **c, d** The relative performance (**c** CPU time and **d** RAM) of the benchmarked aligners grouped by the algorithm used for genome indexing and colored by individual aligners (BWT-FM CPU time vs. Suffix array CPU time: LRT, *p* value = 1.5 × 10^−15^, Hashing memory vs. BWT-FM memory: LRT, *p* value = 2.2 × 10^−3^, BWT-FM memory vs. Suffix Array memory: LRT, *p* value < 2 × 10^−16^). The legend of **d** is the same for **c**, **e**, and **f**. **e** The relative performance (CPU time) of the benchmarked aligners grouped by whether the tool was released before or after long-read technology was introduced (2013) and colored by individual aligners (LRT, *p* value = 3.7 × 10^−11^). **f** The relative performance (CPU time) of the benchmarked aligners grouped by the algorithm used for pairwise alignment and colored by individual aligners (Needleman-Wunsch CPU time vs. Smith-Waterman CPU time: Wald, *p* value = 1.3 × 10^−4^, Needleman-Wunsch CPU time vs. Hamming Distance CPU time: Wald, *p* value = 9.3 × 10^−7^, Needleman-Wunsch CPU time vs. Non-DP Heuristic CPU time: Wald, *p* value = 1.8 × 10^−10^)

To measure the effect of read alignment algorithms on speed of alignment and computational resources, we have compared the running time and memory (RAM) required of eleven read alignment tools when applied to ten real WGS datasets (Fig. 4a, b). We used tools available via the Bioconda package manager [146]. We ran these tools using their default parameters. We randomly selected ten WGS samples from the 1000 Genomes Project. We excluded tools specifically designed for RNA-Seq or BS-Seq. Details on how the tools were installed and ran are provided in Supplementary Note 3.

We found no significant difference in the runtime for BWT-FM tools and hashing-based tools when adjusting for year of publication, chain of seeds, and type of pairwise alignment (Likelihood ratio test (LRT) *p* value = 0.5) (Fig. 4c, Supplementary Table 3, 4). SMALT [69] is an outlier to this observation, and it shows the highest execution time (Fig. 4c) as it uses standard non-accelerated pairwise alignment algorithm (Smith-Waterman algorithm). BWT-FM-based tools did require, on average, 3.8× less computational resources when compared to hashing-based tools, adjusting for year of publication, chain of seeds, and type of pairwise alignment algorithm (LRT *p* value = 2.2 × 10^−3^) (Fig. 4d, Supplementary Table 5, 6). SNAP [81] shows the highest memory footprint (Fig. 4d) as its index exceptionally uses much longer (> 20 bp) seeds compared to most other tools. The default suffix array implemented by LAST [78] requires, on average, 4.38× more running time and 3.58× more computational resources when compared to BWT-FM-based tools (LRT test *p* value = 1.5 × 10^−15^ and < 2 × 10^−16^ for runtime and memory, respectively) (Fig. 4c, d, Supplementary Table 3, 4, 5, 6).

Despite the difference in performance driven by algorithms, we observed an overall improvement (9.2× reduction) in computation time of read alignment over time (s.e. = 0.09; LRT test *p* value = 3.7 × 10^−11^) (Fig. 4e, Supplementary Table 3, 4) but no significant improvement (only 1.57× reduction) of their memory requirements (s.e. = 0.24; LRT *p* value = 0.41) (Supplementary Figure 1, Supplementary Table 5, 6). Usually, the index is created separately for each genome. Some methods incorporate multiple genomes into a single index graph [58, 76, 115], while other methods use a de Bruijn graph for hashing [58, 116]. Although computing the genome index can take up to four hours, it usually needs to be computed only once and is often already precomputed for various species (Supplementary Figure 2). Updating the genome index can create a bottleneck in the analysis, especially for extremely large genome databases. Bloom1-filter-based algorithms promise to provide an alternative way of indexing while preserving faster search times [125, 147].

We surveyed 28 BWT-FM-based tools to compare the popularity of the read alignment algorithms using the number of times the introductory publication has been cited in other papers. Of those, three aligners have accumulated more than 1000 citations per year since release, and 18% of the BWT-FM-based tools have been cited by at least 500 papers per year. In contrast, only two of the 63 hashing-based tools have more than 1000 citations per year, but those two aligners (BLAST [31] and Gapped BLAST [32]) are, by far, the most popular with 2726 and 3143 citations per year, respectively (Fig. 3b). Notably, tools cited more than 500 times per year were among the most effective both in terms of runtime and required computational resources (Supplementary Figure 3).

## Majority of the tools utilize fix length seeding to find the global position of the read in the reference genome

The goal of the second step of read alignment is to find the global position of the read in the reference genome. This step is known as global positioning and uses the generated genome index to retrieve the locations (in the genome) of various seeds extracted from the sequencing reads (Fig. 1c). The read alignment algorithm uses the determined seed locations to reduce the search space from the entire reference genome to only the neighborhood region of each seed location (Supplementary Note 4).

The number of possible locations of a seed in the reference genome is affected by two key factors: the seed length and the seed type. The estimated number of such locations is extremely large for short seeds and can reach tens of thousands for the human genome. The high frequency of short seeds is due to the repetitive nature of most genomes, which creates a high probability of finding the same short seed frequently in a long string of only four DNA letters. A large number of possible locations for short seeds imposes a significant computational burden on read alignment algorithms [148, 149]. Only a few read alignment algorithms examine all the seed locations reported in the location list [102]. Most of the read alignment algorithms apply heuristic devices to avoid examining all the locations of the seed in the reference genome (Fig. 1d, Supplementary Note 4).

Longer seed lengths can help reduce both the number of possible locations of a seed in the reference genome and the number of chosen seeds from each read. These benefits come at the cost of a possible reduction in alignment sensitivity, especially in cases where the mismatches between the read and the genome are located within the seed sequence. To enable increasing the seed length without reducing the alignment sensitivity, seeds can be generated as spaced seeds (Supplementary Note 4 ) [34–37, 139].

The majority of the surveyed alignment algorithms use seeds of fixed length at run time. Some algorithms generate seeds of various lengths [83, 108, 150] in order to reduce the hit frequencies while tolerating mismatches. Varying the seed length or using different types of seed during the same run is often referred to as hybrid seeding [108] and was used by 20 of the 107 surveyed alignment algorithms. The first tool to use variable-length seeds was GMAP [41]. Hybrid seeding with a hash-based index would require the creation of multiple hash tables of the same genome and would require extra computational resources. As a result, the vast majority of tools that use variable-length seeds use a suffix tree indexing technique (BWT-FM or other).

## Majority of the tools utilize Hamming distance and Smith-Waterman to determine similarity between the read and its global positions in the reference genome

The goal of the last step of a read alignment algorithm is to determine regions of similarity between each read and the global positions of each read in the reference genome, which was determined in the previous step. These regions are potentially highly similar to the reads, but read alignment algorithms still need to determine the minimum number of differences between two genomic sequences, the nature of each difference, and the location of each difference in one of the two given sequences. Such information about the optimal location and the type of each edit is normally calculated using a verification algorithm (Fig. 1f) that first verifies the similarity between the query read and the corresponding region in the reference genome. Verification algorithms can be categorized into algorithms based on dynamic programming (DP) [151] and non-DP-based algorithms. The DP-based verification algorithms can be implemented as local alignment (e.g., Smith-Waterman [152]) or global alignment (e.g., Needleman-Wunsch [153]). DP-based verification algorithms can also be implemented as semi-global alignment, where the entirety of one sequence is aligned to one of the ends of the other sequence [108, 109, 117].

The non-DP verification algorithms include Hamming distance [154] and the Rabin-Karp algorithm [155]. When one is interested in finding genetic substitutions, insertions, and deletions, DP-based algorithms are favored over non-DP algorithms. In general, the local alignment algorithm is preferred over global alignment when only a fraction of the read is expected to match with some regions of the reference genome due to, for example, large structural variations [63]. The Smith-Waterman [152] and Needleman-Wunsch [153] alignment algorithms were both first used by FASTA [30, 138] in 1988, which we categorize as “Multiple Methods” (Fig. 3c). Smith-Waterman remains the most popular algorithm and is used by 28.3% of our surveyed tools (Fig. 2). Needleman-Wunsch, in contrast, has only been used by 16.2% of our surveyed tools (Fig. 2). However, if we include the tools which allow for multiple methods, Smith-Waterman represents 38.3% and Needleman-Wunsch represents 26.2% of alignment algorithms used. This trend is due to the fact that 12 of the 13 tools classified as “Multiple Methods” use or allow both Smith-Waterman and Needleman-Wunsch. Non-DP verification using Hamming distance [154] has been the second most popular single technique since used for the first time by RMAP [44] in 2008 (Fig. 3c). There is no significant correlation between the indexing technique used and the pairwise alignment algorithm chosen. Most major indexing techniques are used in conjunction with most pairwise alignments. However, BWT-FM-based aligners do comprise the largest percentage of tools that allow multiple pairwise alignment methods (Fig. 2).

As the number of differences between two sequences is not necessarily equivalent to the sum of the number of differences between the subsequences of these sequences, it is necessary to perform verification for the entire read sequence and the corresponding region in the reference sequence [156]. Existing DP-based algorithms can be inefficient as they require quadratic time and space complexity. Despite more than three decades of attempts to improve their algorithmic implementation, the fastest known edit distance computation algorithm is still nearly quadratic [157]. Some of the read alignment algorithms use DP only for seed chaining, which provides suboptimal alignment calculation [38, 40]. This approach is called sparse DP and is used in C4 [40], conLSH [135], and LAMSA [122]. An alternative way to accelerate the alignment algorithms is by reducing the maximum number of differences that can be detected by the verification algorithm, which reduces the search space of the DP algorithm and shortens the computation time [106, 158–164, 167, 168] (Supplementary Note 5).

We found that tools which use the Needleman-Wunsch [153] algorithm are faster than tools which use other algorithms (faster by 3.57×, 4.14×, and 6.7× and Wald test *p* values 9.3 × 10^−7^, 1.8 × 10^−10^, and 1.3 × 10^−4^ for Hamming distance, non-DP heuristics, and SW algorithms, respectively) (Fig. 4f, Supplementary Table 3), adjusting for publication year, seed chaining, and indexing method. Despite the overall longer runtime of Hamming distance-based methods, the latest hashing-based tools (e.g., HISAT2 [133]) provide a comparable running time with the fastest Needleman-Wunsch-based tools. We also found significant differences in the amount of computational resources required by read alignment tools using different pairwise alignment algorithms after adjusting for publication year, type of seed, and indexing method (LRT; *p* value = 0.04) (Supplementary Figure 4, Supplementary Table 6). Notably, the algorithms with the smallest computational footprints use various types of pairwise alignment algorithms.

## Influence of long-read technologies on the development of novel read alignment algorithm

Alignment of the long reads produced by modern long-read technologies [16, 136, 169] provides a unique possibility to discover previously undetectable structural variants [16, 170, 171]. Long reads also improve the construction of an accurate hybrid de novo assembly [16, 172], in cases where long and short reads are suffix-prefix overlapped, or in cases where reads are aligned using pairwise alignment algorithms, to construct an entire assembly graph. This is helpful when a reference genome is either unavailable [173, 174] or is complex and contains large repetitive genomic regions [175].

Existing long-read alignment algorithms still follow the three-step-based approach of short-read alignment. Some long-read alignment tools even divide every long read into short segments (e.g., 250 bp), align each short segment individually, and determine the mapping locations of each long read based on the adjacent mapping locations of these short segments [123, 127]. Some long-read alignment tools use hash-based indexing [110, 120, 176], while others use BWT-FM indexing [54, 98, 177]. The major challenge with the long-read alignment algorithms is dealing with large sequencing errors and a significantly large number of short seeds extracted from each long or ultra-long read [178]. Thus, the most recently developed long-read alignment algorithms require heuristically extracting fewer seeds per read length when compared to those extracted from short reads. Instead of creating a hash table for the full set of seeds, recent long-read alignment algorithms find the minimum representative set of seeds from a group of adjacent seeds within a genomic region. These representative seeds are called minimizers [179, 180] and can also be used to compress genomic data [181] or taxonomically profile metagenomic samples [182]. Long-read alignment algorithms [119, 124, 183] that use hashed minimizers as an indexing technique provide a faster alignment process compared to other algorithms that use conventional seeding or BWT-FM. They also provide a significantly faster (> 10×) indexing time (Supplementary Table 1). However, their accuracy degrades with the use of short reads as they process a fewer number of seeds per short read [124].

**Box 1. Advantages and limitations of short- versus long-read alignment algorithms**

**Table:** 

• Error rate. The error rate of modern short-read sequencing technologies is smaller than that of modern long-read technologies.	
• Genome coverage. Throughput (i.e., the number of reads) of modern short-read sequencing technologies is higher than that of modern long-read technologies.	
• Global position. Determine a global position of the read by identifying the starting position or positions of the reads in the reference genome. This step is ambiguous with short reads, as the repetitive structure of the human genome causes such reads to align to multiple locations of the genome. In contrast, long reads are usually longer than the majority of repeat regions and are aligned to a single location in the genome.	
• Local pairwise alignment. After determining the global position of each read, the algorithms map all bases of the read to the reference segments, located at these global positions, in order to account for indels. Due to the smaller error rate of short-read technologies, it is usually easier to perform local alignment on short reads than on long ones.	
• Genomic variants. Single-nucleotide polymorphisms (SNPs) are easy to detect using short reads when compared to long reads due to the lower error rate and higher coverage of short-read sequencing technologies. Structural variants (SVs) are easy to detect with long reads, which span the entire SV region. Current long-read-based tools [184] are able to detect deletions and insertions with high precision. The sparse coverage of long reads may lower the sensitivity of detection.	

## Read alignment across various domains of biological research

We discuss the challenges and the features of these algorithms that are specific to the various domains of modern biological research. Often the domain-specific alignment problem can be solved by creating a novel tool from scratch or wrapping the existing algorithms into a domain-specific alignment tool (Supplementary Figure 5 and 6). Additionally, longer reads make the read alignment problem similar across areas of biological research. For example, tools recently designed to align long reads can handle both DNA and RNA-Seq reads [131].

## RNA-Seq alignment

RNA sequencing is a technique used to investigate transcriptomics by generating millions of reads from a collection of human alternative spliced isoform transcripts, referred to as a transcriptome [185]. RNA-Seq has been widely used for gene expression analysis as well as splicing analysis [14, 185, 186]. However, the alignment of RNA sequencing reads needs to overcome additional challenges when mapping the reads originating from human transcriptome onto the reference genomes. Those challenges arise due to differences between the human transcriptome and the human genome; these differences define a subset of alignment problems known as *spliced alignment*. Spliced alignment requires that the one takes into account reads spanning over large gaps caused by spliced out introns [185]. Reads spanning only a few bases across the junctions can be easily aligned to an adjacent intron or aligned in a wrong location, making the accurate alignment more difficult [14, 185].

Several spliced alignment tools have been developed to address this issue and align RNA-Seq reads in a splicing-aware manner (Table 1 and Fig. 1c). Hashing is the most popular technique among RNA-Seq aligners (Supplementary Figure 7). This is even more evident if we remove the RNA-Seq aligners that are wrappers of existing DNA-Seq alignment methods (Supplementary Figure 5). Over 60% of the RNA-Seq aligners which are wrappers of existing DNA-Seq alignment methods use Bowtie or Bowtie2 (Supplementary Figure 5). When considering only stand-alone RNA-Seq aligners, the number of aligners using hashing more than doubles the number of aligners using an FM index (Supplementary Figure 8).

The most popular tool based on the number of citations was TopHat2 [105] (Table 1). TopHat2 uses Bowtie2 to align reads that may span more than one exon by splitting the reads into smaller segments and stitching the segments together to form a whole read alignment. The stitched read alignment spans a splicing junction on the human genome. This method allows identification of the splicing junction without transcriptome alignment. A more recent tool, HISAT2, uses a hierarchical indexing algorithm that leverages the Burrows-Wheeler Transform and Ferragina-Manzini index to align parts of reads and extend the alignment [115]. Another popular method, RNA-Seq aligner—called STAR—utilizes suffix arrays to identify a maximal mappable prefix, which is used as seeds or anchors, and stitch together the seeds that aligned within the same genomic window [104]. Although those tools can detect splicing junctions within their algorithm, it is possible to supply known gene annotation to increase the accuracy of a spliced alignment. The alignment accuracy, measured by correct read placement, can be increased 5–10% by supplying known gene annotations [14, 185]. HISAT2 and STAR are able to align the reads accurately with or without a splicing junction [14]. Furthermore, the discovery and quantification of novel splicing junctions can be significantly improved using two passes in STAR, which generates a list of possible junctions in the first pass and identifies aligning reads leveraging the junctions in the second pass [187]. While spliced alignment can provide an important splicing junction information, those tools require intensive computational resources [14].

To align RNA-Seq reads onto the transcriptome reference instead of the genome reference, regular DNA aligners are typically used. Mapping to the transcriptome is usually performed to estimate expression levels of genes and alternatively spliced isoforms by assigning reads to genes and alternatively spliced isoforms [104, 188]. Since many alternatively spliced isoforms share exons, which are usually longer than the short reads, probabilistic models are used as it is impossible to uniquely assign reads to the isoform transcripts [189].

Alternatively, one can avoid computationally expensive alignment and perform pseudo-alignment, such as Kallisto [104] and Salmon [187]. Kallisto [190] uses transcriptome de Bruijn graph as an index where its nodes are seeds. Kallisto determines the locations of each input read by matching seeds extracted from reads with the seeds of the index without performing sequence alignment. Kallisto also exploits the structure of the de Bruijn graph to avoid examining more than a few seeds located at the same graph’s path (between two junctions). This reduces the number of seed lookups in the index and hence reduces expensive memory accesses.

In contrast, Salmon [190, 191] can optionally perform either pseudo-alignment or read alignment. Salmon approximates the locations of each input read by building a hashing index in conjunction with a suffix array index. The seeds extracted from each read are looked up in the hash table and then the suffix array provides all suffixes of the reference genome containing the matched seed. Similar to Kallisto, Salmon tries to reduce the number seed lookups by finding the longest subsequence of the read that exactly matches the reference suffixes and excluding these regions from seed lookups.

In contrast to regular alignment algorithms, pseudo-alignment algorithms [190, 191] are unable to provide the precise alignment position of the read in the genome nor alignment profile (e.g., CIGAR string). Instead, pseudo-alignment algorithms assign the reads to a corresponding gene and/or alternatively spliced isoform. Usually, such information can be sufficient to accurately estimate gene expression levels of the sample [192]. A higher sequencing depth is demonstrated to improve the accuracy of Salmon and decreases the accuracy of Kallisto, as only Salmon exploits abundance information of each isoform to assist the seed matching [188].

## Metagenomic alignment

Metagenomics is a technique used to investigate the genetic material in human or environmental microbial samples by generating millions of reads from the microbiome—a complex microbial community residing in the sample. Metagenomic data often contains an increased number of reads required to be aligned against more than hundreds of thousands of microbial genomes. For example, as of July 2018, the total number of nucleotides in NCBI’s collection of bacterial genomes measures over 204 times the number of nucleotides present in the Genome Reference Consortium Human Build 38 (Supplementary Note 6). The increased number of reads and the size of reference databases pose unique challenges to existing alignment algorithms when applied to metagenomics studies.

In targeted gene sequencing studies, such as those that sequence portions of the 16S ribosomal RNA of prokaryotes or internally transcribed spacers (ITS) of eukaryotes, a number of task-specific aligners are utilized to identify the origin of candidate reads or to perform homology searches. For example, Infernal [193] utilizes profile hidden Markov models to perform alignment based on RNA secondary structure information. Multiple sequence aligners are also utilized in metagenomic analysis pipelines such as QIIME [194], Mothur [195], and Megan [195, 196]. For example, NAST [195–197] and PyNAST [198] use 7-mer seeds and a BLAST alignment that is then further refined using a bidirectional search to handle indels. Similarly, MUSCLE [198, 199] uses an initial distance estimation based on *k*-mers and proceeds through a progressively constructed hierarchical guide tree while optimizing a log expectation for multiple sequence alignment [199].

For untargeted whole genome shotgun (WGS) metagenomic studies, the task of identifying the genomic or taxonomic origin of sequencing reads (referred to as “fragment recruitment” or “taxonomic read binning”) is even more difficult, individual reads can originate from multiple organisms due to shared homology or horizontal gene transfer and reads may originate from previously unsequenced organisms. This has sparked the development of a variety of tools [200] which aim to identify the presence and relative abundance of taxa or organisms present in a metagenomic sample via a reference-free and/or alignment-free fashion (referred to as “taxonomic profiling”). Similar in spirit to RNA-Seq alignment, these tools avoid computationally expensive base-level alignment and perform pseudo-alignment or multiple types of *k*-mer matching to detect the presence of organisms in a metagenomic sample [182, 201, 202], as well as use minimizers to reduce computational time [182].

Other approaches handle growing reference database sizes by aligning reads onto a reduced reference database, sometimes composed of marker microbial genes that are present in specific taxa. Reads mapping to those genes can be used to determine the presence of specific taxa in a sample [203]. Such tools typically use existing DNA alignment algorithms (e.g., MetaPhlAn [203] uses the Bowtie2 aligner).

Even with the development of these new metagenomic tools, existing read alignment tools (e.g., MOSAIK, SOAP, and BWA) are still used for fragment recruitment purposes [204]. However, the use of existing read alignment tools for metagenomics carries a significant computational burden and is identified as the main bottleneck in the analysis of such data. This major limitation suggests the need for the development of alignment tools capable of handling the increased number of reads and reference genomes seen in such studies [205].

Metagenomics studies are also capable of functional annotation of microbiome samples by aligning the reads to genes, gene families, protein families, or metabolic pathways. Protein alignment is beyond the scope of this manuscript, but many of the algorithmic approaches previously discussed are utilized for functional annotation [204, 206]. For example, RAPSearch2 [204, 206] uses a collision-free hash table based on amino acid 6-mers. The protein aligner DIAMOND [207] utilizes a spaced-seed-and-extend approach based on a reduced alphabet and unique indexing of both reference and query sequences. Indexing of both the reference *and* the query reads provides multiple orders of magnitude in speed improvements over older tools (such as BLASTX) at the cost of increased memory usage. Recently, MMseqs2 [205] utilizes consecutive, similar *k*-mer matches to further improve the speed of protein alignment.

## Viral quasispecies alignment

RNA viruses such as human immunodeficiency virus (HIV) are highly mutable, with the mutation rates being as high as 10^−4^ per base per cell [208] allowing such viruses to form highly heterogeneous populations of closely related genomic variants commonly referred to as quasispecies [209]. Rare genomic variants, which are a few mutations away from the major strain, are often responsible for immune escape, drug resistance, viral transmission, and increase of virulence and infectivity of the viruses [210, 211]. Massively parallel sequencing techniques allow for sampling of intra-host viral populations at high depth and provide the ability to profile the full spectra of viral quasispecies, including rare variants.

Similar to other domains, accurate read alignment is essential for assembling viral genomic variants including the rare ones. Aligning reads that originated from heterogeneous populations of closely related genomic variants to the reference viral genome give rise to unique challenges for existing read alignment algorithms. For example, read alignment methods should be extremely sensitive to small genomic variations while being robust to artificial variations introduced by sequencing technologies. At the same time, the genetic difference between viral quasispecies of different hosts is usually substantial (unless they originated from the same viral outbreak or transmission cluster), which makes the application of predefined libraries of reference sequences for viral read alignment problematic or even impossible.

Currently, viral haplotyping tools [212, 213] and variant calling tools [214, 215] frequently rely on existing independent alignment tools. While viral samples contain several distinct haplotypes, the read alignment tools such as BWA [145] and BowTie [216] can only map reads to a single reference sequence. Since certain haplotypes may be further or closer to the reference sequence, the reads emitted by such haplotypes may have different mapping quality. Some tools re-align reads to the consensus sequence instead of keeping the original alignment to the reference. Nevertheless, even alignment to the perfect reference or consensus sequence can reject perfectly valid short reads because of multiple mismatches. Rejection of such reads may cause loss of rare haplotypes and mutations. Systematic sequencing errors (such as homopolymer errors) frequently cause alignment errors. Although the sequencing error rate, both systematic and random, is comparatively low, such errors can be more frequent than the rarest variants. The alignment errors caused by sequencing errors may cause drastic sensitivity and reduction in specificity of haplotyping and variant calling methods (Supplementary Figure 9).

## Aligning bisulfite-converted sequencing reads

Bisulfite-converted sequencing is a technique used to sequence methylated fragments [217, 218]. During sequencing, most of the cytosines (C) in the reads become thymines (T). Since every sequenced T could either be a genuine genomic T or a converted C, special techniques are used to map those reads [219]. Some tools substitute all C in reads with wildcard bases, which can be aligned to C or T in the reference genome [37, 52], while other tools substitute all C by T in all reads and reference and work with a three-letter alphabet aligning to a C-to-T-converted genome [77, 96]. Unlike RNA-Seq aligners, FM index was the most popular technique among BS-Seq aligners (Supplementary Figure 10). One-third of the surveyed BS-Seq aligners were wrappers of existing DNA-Seq alignment methods (Supplementary Figure 6), with all three of those wrapping Bowtie or Bowtie2 (Supplementary Figure 6). As a result, when considering only stand-alone BS-Seq aligners, the numbers of aligners using each indexing algorithm become extremely similar (Supplementary Figure 11).

## Other domains

Other domains requiring specialized alignment include B and T cell receptor repertoire analysis. The repertoire data is generated using targeter repertoire sequencing protocols, known as BCR- or TCR-Seq. For example, tools designed to align reads to the V(D)J genes use combinations of fast alignment algorithms and more sensitive modified Smith-Waterman and Needleman-Wunsch algorithms [182, 220, 221].

## Discrepancies between the reads and the reference may reveal the historical errors in the reference assembly

Genome sequencing datasets, especially those generated with long reads, provide a unique perspective to reveal errors in the reference assemblies (e.g., human reference genome) based on the discrepancies between the reads and the reference sequence. References and reads (e.g., resequencing data) are often produced using different technologies, and there are usually disagreements between references and reads that produce mapping errors. Similarly, some of these errors also come from the errors in the reads used for assembly, collapsed/merged duplications/repeats, and heterozygosity. For example, a study for structural variation discovery led to the identification of incorrectly inverted segments in the reference genome [222]. Similarly, Dennis et al. [223] characterized a duplicated gene that was not represented accurately because it collapsed in the reference genome. Therefore, using the most recent version of a reference genome is always the best practice, as demonstrated by an analysis of the latest version of the human genome [223, 224].

Structural errors in the reference genomes can be found and corrected by using various orthogonal technologies such as mate-pair and paired-end sequencing [225, 226], optical mapping [227], and linked-read sequencing [228]. Smaller-scale errors (i.e., substitutions and indels) can also be corrected using assembly polishing tools such as Pilon, which employs short-read sequencing data [229]. However, long reads are more powerful in detecting and correcting errors due to the fact that they can span the most common repeat elements. Long-read-based assembly polishers include Quiver [230] that uses Pacific Biosciences data, Nanopolish [231] that uses Nanopore sequencing, and Apollo [232] that can use read sets from any sequencing technology to polish large genomes. Additionally, more modern long-read genome assemblers, such as Canu [233], include built-in assembly polishing tools.

## Discussion

Rapid advances in sequencing technologies shaped the landscape of modern read alignment algorithms leading to today’s diverse array of alignment methods. Those technological changes rendered some read alignment algorithms irrelevant—yet provide context for the development of new tools better suited for modern next-generation sequencing data. The development of alignment algorithms is shaped not only by the characteristics of sequencing technologies but also by the specific characteristics of the application domain. Often different biological questions can be answered using similar bioinformatics algorithms. For example, BLAT [38, 234], a tool that was originally designed to map EST and Sanger reads, is now used to map the assembled contigs to the reference genome [234]. Specific features of various domains of biological research, including whole transcriptome, adaptive immune repertoire, and human microbiome studies, confront the developer with a choice of developing a novel algorithm from scratch or adjusting existing algorithms.

In general, the read alignment problem is extremely challenging due to the large size of analyzed datasets and numerous technological limitations of modern sequencing platforms. A modern read aligner should not only be able to maintain a good balance between speed and memory usage but also be able to preserve small and large genetic variations. It should be capable of tackling numerous technological limitations and changes, ultimately inducing rapid evolution of sequencing technologies such as constant growth of read length and changes in error rates. In general, determining an accurate global position of the read in the reference genome provides no guarantee that accurate local pairwise alignment can be found. This is especially challenging for the error-prone long reads, where determining the accurate global position of the read in the reference genome is usually easy, but local pairwise alignment represents a substantial challenge due to a high error rate.

This review not only provides an understanding of the basic concepts of read alignment, its limitations, and how they are mitigated but also helps inform its future directions in read alignment development. We believe the future is bright for read alignment algorithms, and we hope that the many examples of read alignment algorithms presented in this work inspire researchers and developers to enhance the field of computational genomics by accurate and scalable tools.

## Supplementary Information

**Additional file 1.** Supplementary tables 1-6; supplementary Figures 1-11; supplementary notes 1-6; supplementary materials.

**Additional file 2.** Review history.

## Data Availability

All data and code required to produce the figures contained within this text are freely available on GitHub: https://github.com/Mangul-Lab-USC/review.technology.dictates.algorithms.
