# Supplementary material for: Technology dictates algorithms: recent developments in read alignment
Source: Genome Biol. 2021 Aug 26;22:249. doi: 10.1186/s13059-021-02443-7 (PMC8390189; doi:10.1186/s13059-021-02443-7)
Supplement: Supplementary file 1 — Additional file 1. Supplementary tables 1-6; supplementary Figures 1-11; supplementary notes 1-6; supplementary materials. [file 13059_2021_2443_MOESM1_ESM.docx]

{Supplementary Information}

**Supplementary Table 1. Genome index size across three read alignment tools.**

| Tool | Version | Index Size | Indexing Time |
| --- | --- | --- | --- |
| mrFAST | 2.2.5 | 16.5 GB | 1202 seconds |
| minimap2 | 0.12.7 | 7.2 GB | 200 seconds |
| BWA-MEM | 0.7.17 | 4.7 GB | 2998 seconds |

**Supplementary Table 2. Tools available in the bioconda package manager. Here we have included only the tools that are primarily built for DNA read alignment.**

| Software tool | Version | Publication | Conda command |
| --- | --- | --- | --- |
| Bowtie2 | 2.2.5 | [^1^](https://paperpile.com/c/c9hsou/62YND) | conda install -c bioconda bowtie2 |
| Bowtie | 0.12.7 | [^2^](https://paperpile.com/c/c9hsou/QwjKW) | conda install -c bioconda bowtie |
| BWA | 0.7.17 | [^3^](https://paperpile.com/c/c9hsou/EfcuR) | conda install -c bioconda bwa |
| GSNAP | 2018-03-25 | [^4^](https://paperpile.com/c/c9hsou/wuskL) | conda install -c compbiocore gsnap |
| HISAT2 | 2.1.0 | [^5^](https://paperpile.com/c/c9hsou/0y1S) | conda install -c bioconda hisat2 |
| LAST | 963 | [^6^](https://paperpile.com/c/c9hsou/NVhup) | conda install -c bioconda last |
| minimap2 | 2.12-r827 | [^7^](https://paperpile.com/c/c9hsou/pylIM) | conda install -c bioconda minimap2 |
| RMAP | 2.1 | [^8^](https://paperpile.com/c/c9hsou/Aad1r) | conda install -c bioconda rmap |
| SMALT | 0.7.6 | [^9^](https://paperpile.com/c/c9hsou/BKuwz) | conda install -c bioconda smalt |
| SNAP | 1.0beta.23 | [^10^](https://paperpile.com/c/c9hsou/ZyN37) | conda install -c bioconda snap-aligner |
| Subread | v1.6.2 | [^11^](https://paperpile.com/c/c9hsou/H0tLp) | conda install -c bioconda subread |

**Supplementary Table 3. Fixed effect size estimates, standard errors (se), test statistics, and p-values for the effect of the listed variables on the expected CPU run time.** The parameters were estimated using the Gamma generalized linear mixed model in Equation (1). “Variable name: Level 1 vs Level 2” indicates that Level 1 is the reference level and the coefficient quantifies the increase / decrease in expected CPU run time for Level 2 over Level 1.

|  | Estimate | se | t stat | p-value |
| --- | --- | --- | --- | --- |
| Intercept | 0.19 | 0.23 | 0.81 | 4.2e-01 |
| Year of publication | -0.7 | 0.09 | -7.96 | 1.7e-15 |
| Chain of seeds: No vs Yes | 1.45 | 0.19 | 7.46 | 8.8e-14 |
| Pairwise alignment: NW vs HD | 1.37 | 0.28 | 4.91 | 9.3e-07 |
| Pairwise alignment: NW vs Non-DP Heuristic | 1.22 | 0.19 | 6.37 | 1.8e-10 |
| Pairwise alignment: NW vs SW | 0.78 | 0.2 | 3.83 | 1.3e-04 |
| Indexing: hashing vs BWT-FM | -0.11 | 0.16 | -0.68 | 5.0e-01 |

**Supplementary Table 4. Likelihood ratio test p-values for the effect of the listed variables on the expected CPU run time.** The parameters (Supplementary Table 3) were estimated using the Gamma generalized linear mixed model in Equation (1). The null Gamma generalized linear mixed model is generated as in Equation (1), but without the variable of interest. Additionally, we ran one LRT between BWT-FM tools and LAST, an aligner that does not use BWT and the FM-index by default.

| Variable of Interest | LRT p-value |
| --- | --- |
| Indexing | 5.0e-01 |
| Year of publication | 3.7e-11 |
| Pairwise alignment | 3.7e-08 |
| BWT-FM vs LAST | 1.5e-15 |

**Supplementary Table 5. Fixed effect size estimates, standard errors (se), test statistics, and p-values for the effect of the listed variables on the expected RAM usage.** The parameters were estimated using the Gamma generalized linear mixed model in Equation (2). “Variable name: Level 1 vs Level 2” indicates that Level 1 is the reference level and the coefficient quantifies the increase / decrease in expected RAM usage for Level 2 over Level 1.

|  | Estimate | se | t stat | p-value |
| --- | --- | --- | --- | --- |
| Intercept | 3.41 | 0.51 | 6.67 | 2.2e-02 |
| Year of publication | -0.21 | 0.24 | -0.85 | 4.8e-01 |
| Chain of seeds: No vs Yes | -0.5 | 0.51 | -0.99 | 4.3e-01 |
| Pairwise alignment: NW vs HD | -1.12 | 0.73 | -1.53 | 2.7e-01 |
| Pairwise alignment: NW vs Non-DP Heuristic | 0.2 | 0.5 | 0.4 | 7.3e-01 |
| Pairwise alignment: NW vs SW | -1.11 | 0.54 | -2.06 | 1.8e-01 |
| Indexing: hashing vs BWT-FM | -1.51 | 0.44 | -3.43 | 7.6e-02 |

**Supplementary Table 6. Likelihood ratio test p-values for the effect of the listed variables on the expected RAM usage.** The parameters (Supplementary Table 5) were estimated using the Gamma generalized linear mixed model in Equation (2). The null Gamma generalized linear mixed model is generated as in Equation (2), but without the variable of interest. Additionally, we ran one LRT between BWT-FM tools and LAST, an aligner that does not use BWT and the FM-index by default.

| Variable of Interest | LRT p-value |
| --- | --- |
| Indexing | 2.2e-03 |
| Year of publication | 4.1e-01 |
| Pairwise alignment | 3.9e-02 |
| BWT-FM vs LAST | 3.2e-77 |


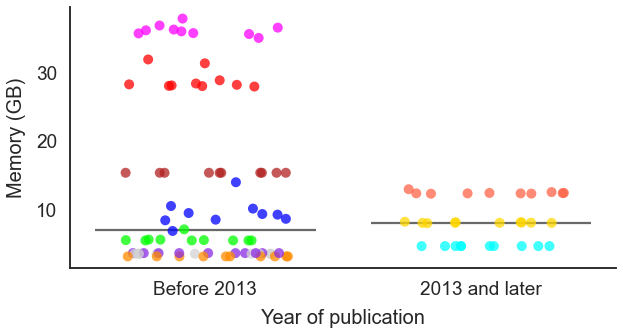


**Supplementary Figure 1. The effect of year of publication on computational resources.** The relative performance (RAM) of the benchmarked aligners grouped by whether the tool was released before or after long read technology was introduced (2013) and colored by individual aligners.


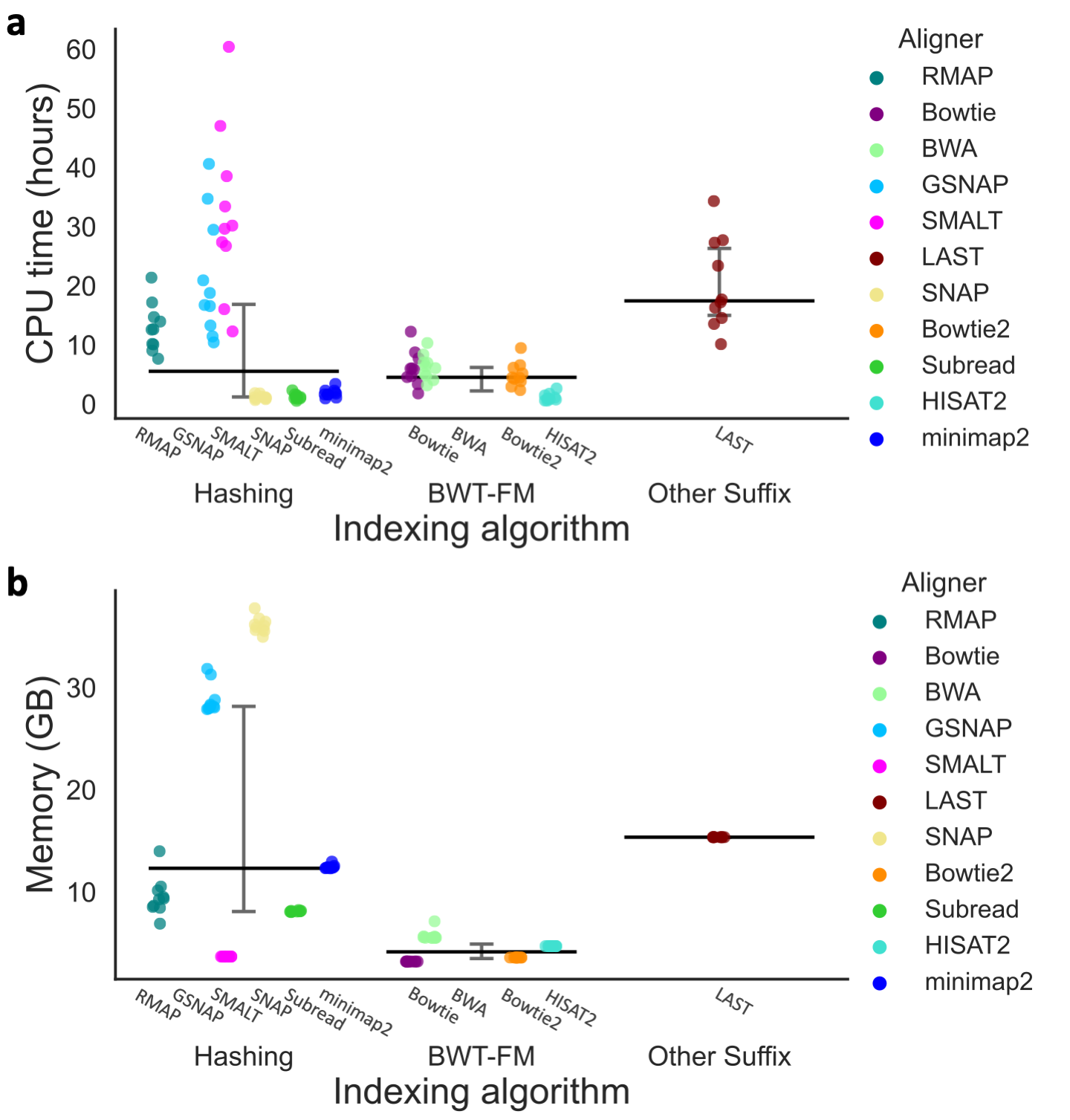


**Supplementary Figure 2. Relative performance of human genome indexing performed by various read alignment tools.** Tools are grouped based on the type of algorithm used for genome indexing. Tools are ordered from oldest (segemehl, 2009) to newest (HISAT2, 2019). (a) CPU time (b) RAM.

**
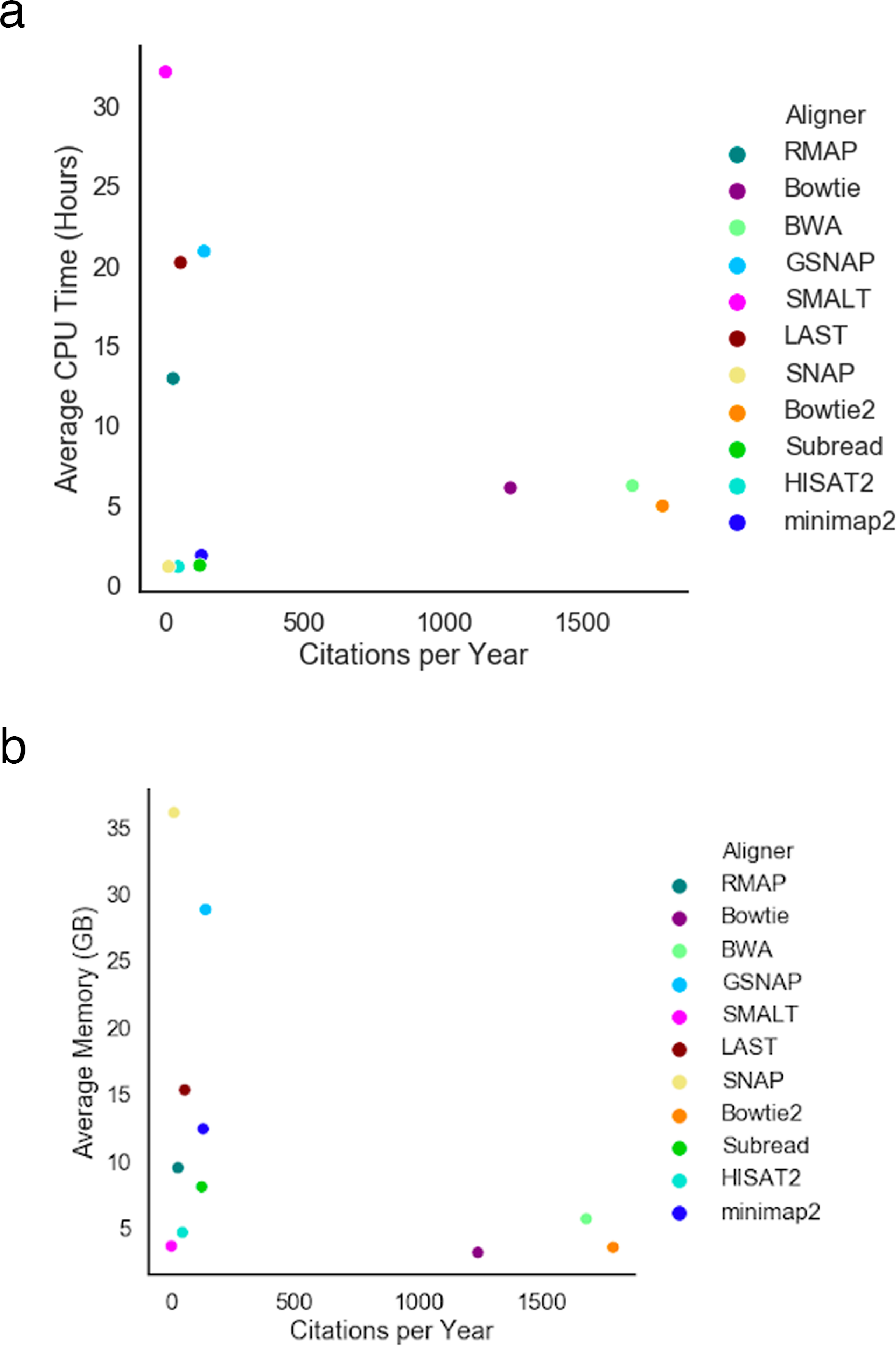
**

**Supplementary Figure 3. Average relative performance of various read alignment tools plotted against the number of citations the tool’s corresponding paper has received yearly since being published.** Tools are ordered from oldest (RMAP, 2008) to newest (minimap2, 2019). (a) CPU time. (b) RAM.


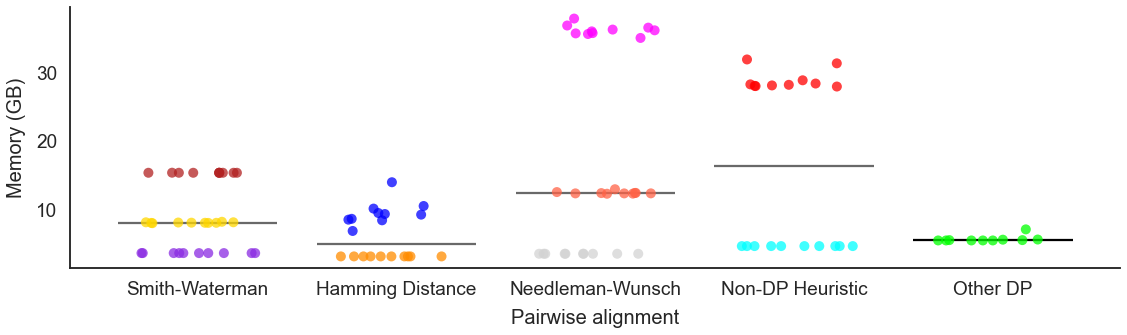


## **Supplementary Figure 4. The effect of pairwise alignment algorithms on computational resources.** The relative performance (RAM) of the benchmarked aligners grouped by the algorithm used for pairwise alignment and colored by individual aligners.

**
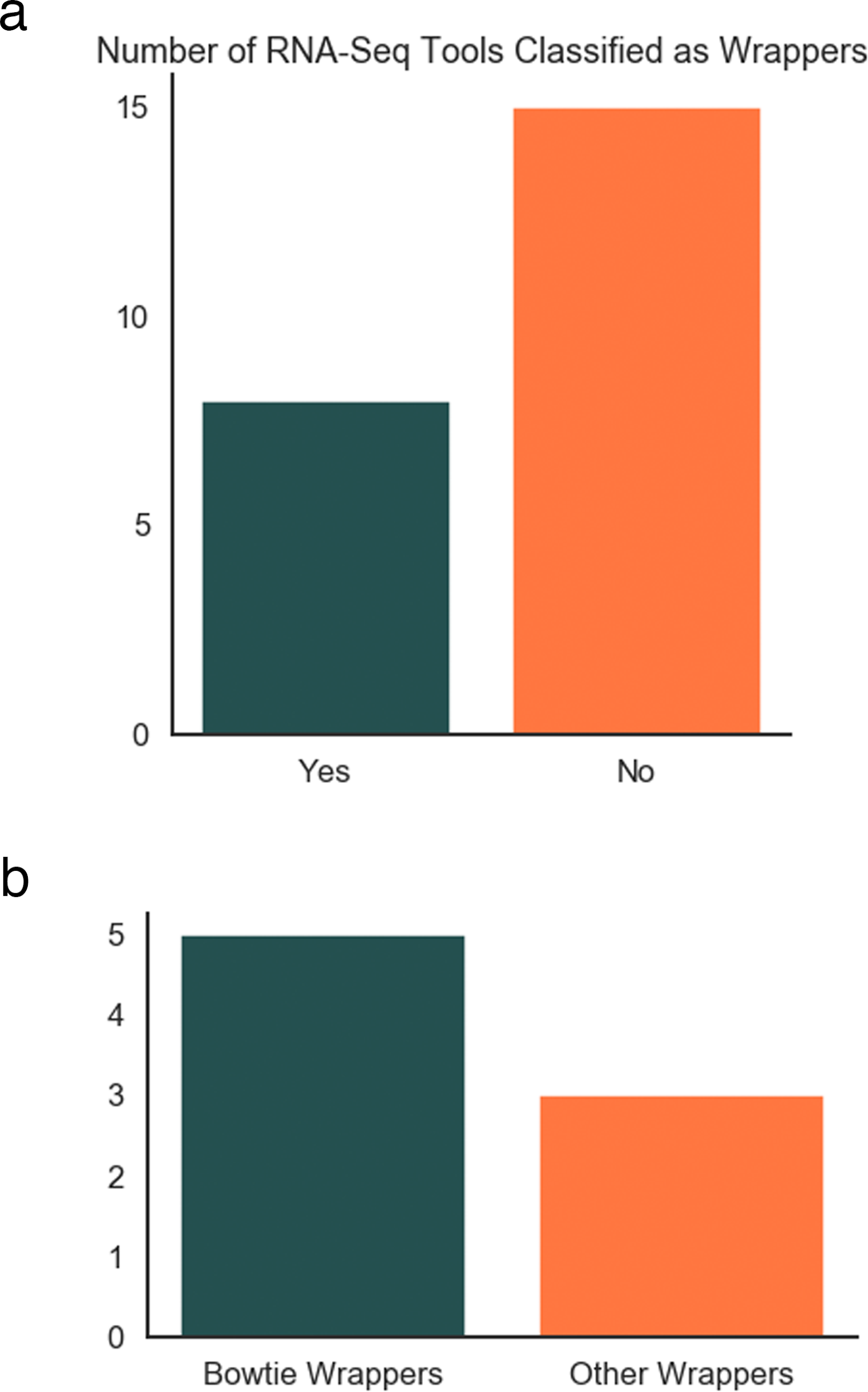
**

**Supplementary Figure 5.** (a) Bar chart showing the number of surveyed RNA-Seq tools which are wrappers of existing DNA-Seq aligners tools. (b) Bar chart showing the number of surveyed RNA-Seq tools which are wrappers of Bowtie or Bowtie2.


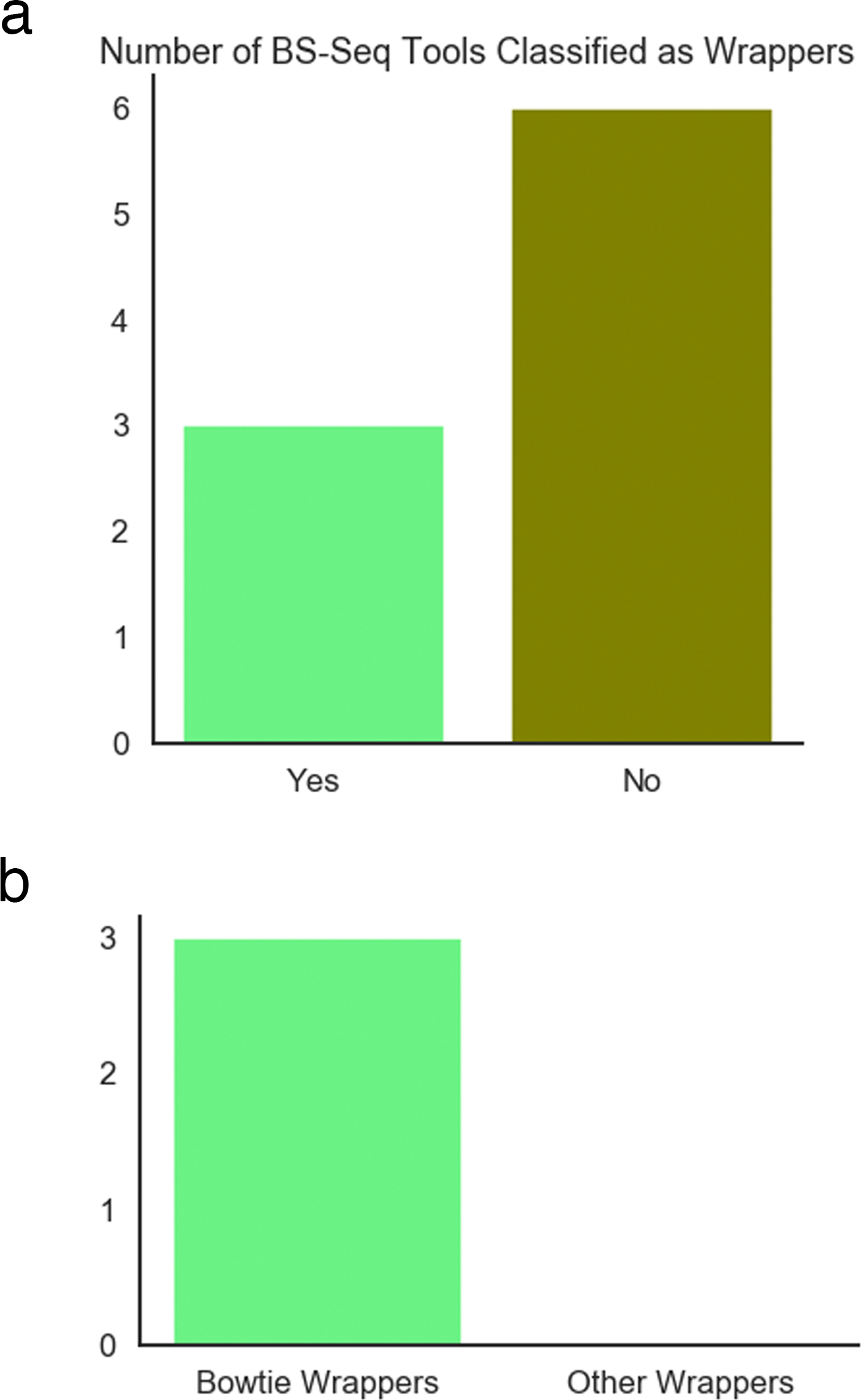


**Supplementary Figure 6.** (a) Bar chart showing the number of surveyed BS-Seq tools which are wrappers of existing DNA-Seq aligners tools. (b) Bar chart showing the number of surveyed BS-Seq tools which are wrappers of Bowtie or Bowtie2.


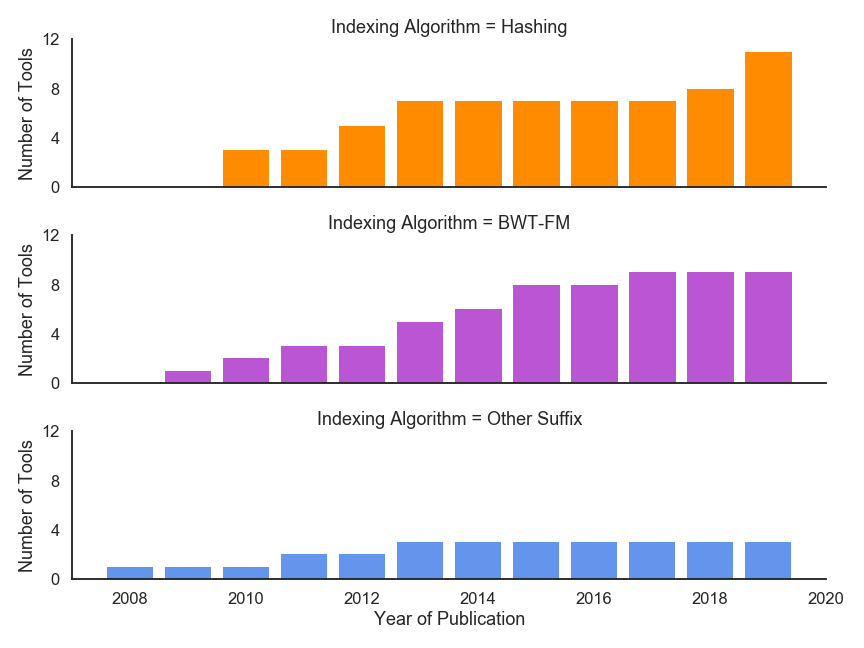


**Supplementary Figure 7. Histogram showing the cumulation of surveyed RNA-Seq tools over time separated by the algorithm used for genome indexing.** This includes both stand alone RNA-Seq tools and wrappers of existing DNA-Seq alignment tools.


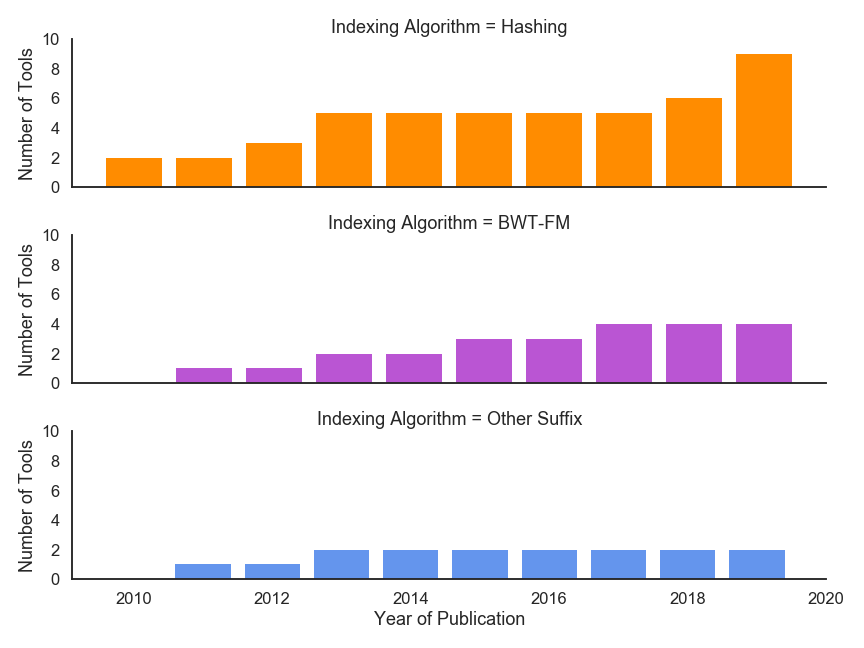
**Supplementary Figure 8. Histogram showing the cumulation of surveyed RNA-Seq tools over time separated by the algorithm used for genome indexing.** Only stand alone RNA-Seq aligners tools are included (not the wrappers of existing DNA-Seq aligners).


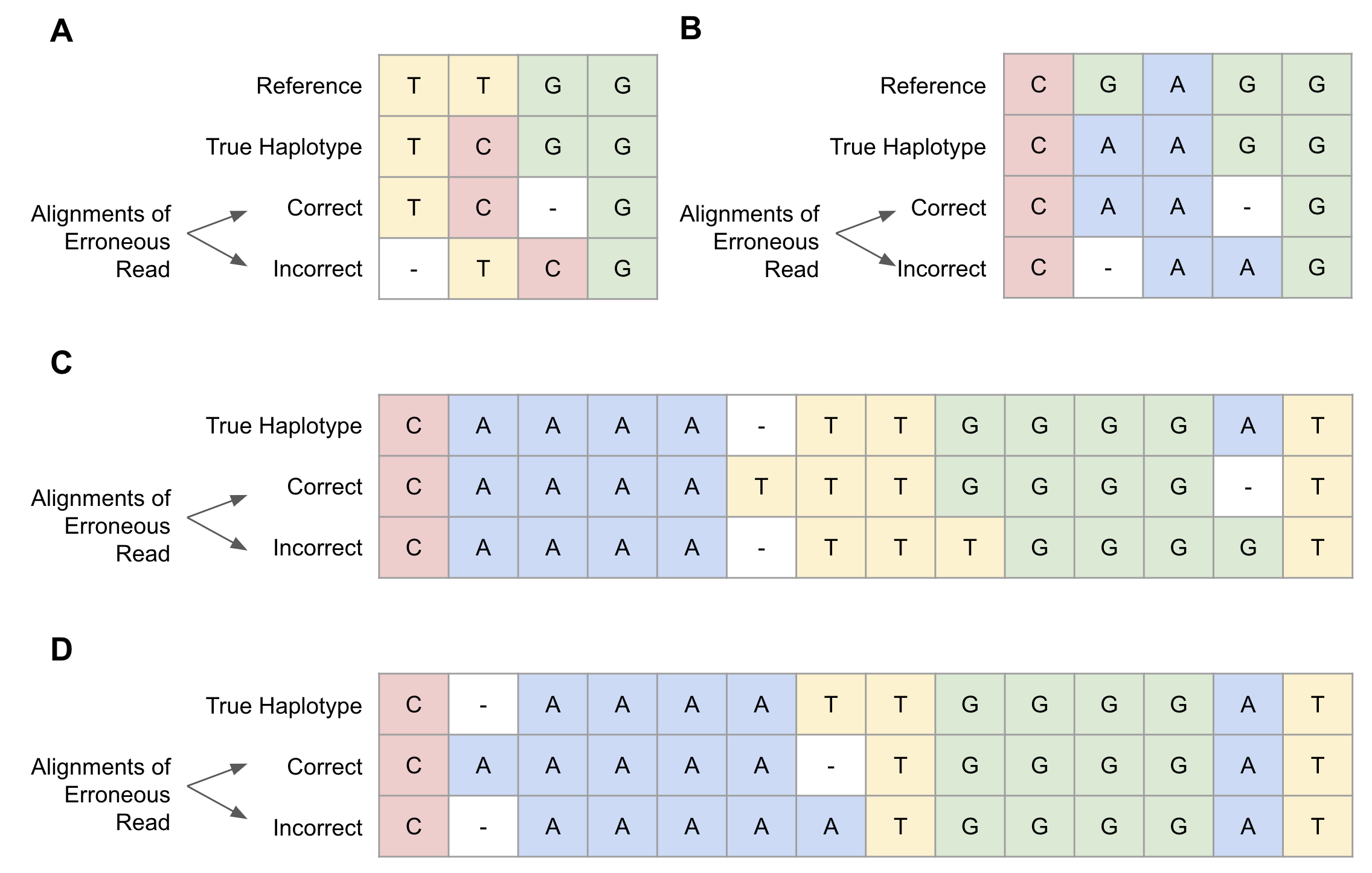


**Supplementary Figure 9. Examples of erroneous alignments of Influenza A virus PacBio sequencing dataset**[^12^](https://paperpile.com/c/c9hsou/3upY4)**.** True viral haplotypes contain deletions that are inconsistently aligned to the reference. Such inconsistencies cause erroneous single-position shifts in the alignment, which in turn results in discovery of false positive single nucleotide variations. (A) and (B) Reads come from a true haplotype with the deletion with respect to the reference. Using BWA scoring method, the reads have two different alignments with the optimal score, but only the first alignment is correct. (C) and (D) Correct read alignment with the homopolymer errors should introduce an insertion and a deletion instead of “optimal” two mismatches.


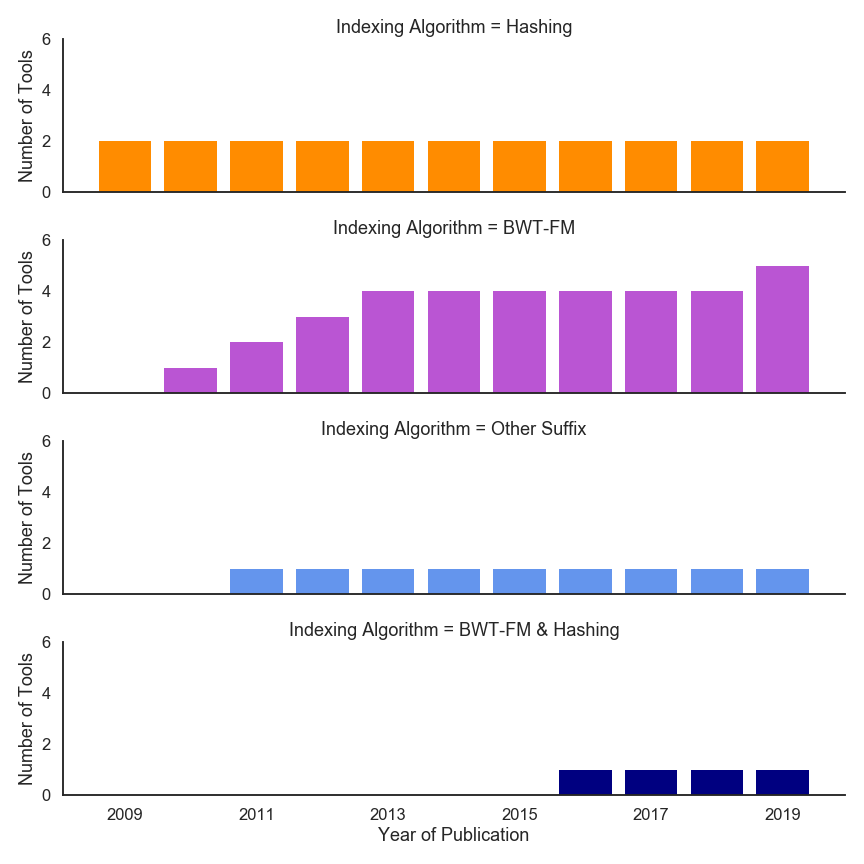


**Supplementary Figure 10. Histogram showing the cumulation of surveyed BS-Seq tools over time separated by the algorithm used for genome indexing.** This includes both stand alone BS-Seq tools and wrappers of existing DNA-Seq alignment tools.


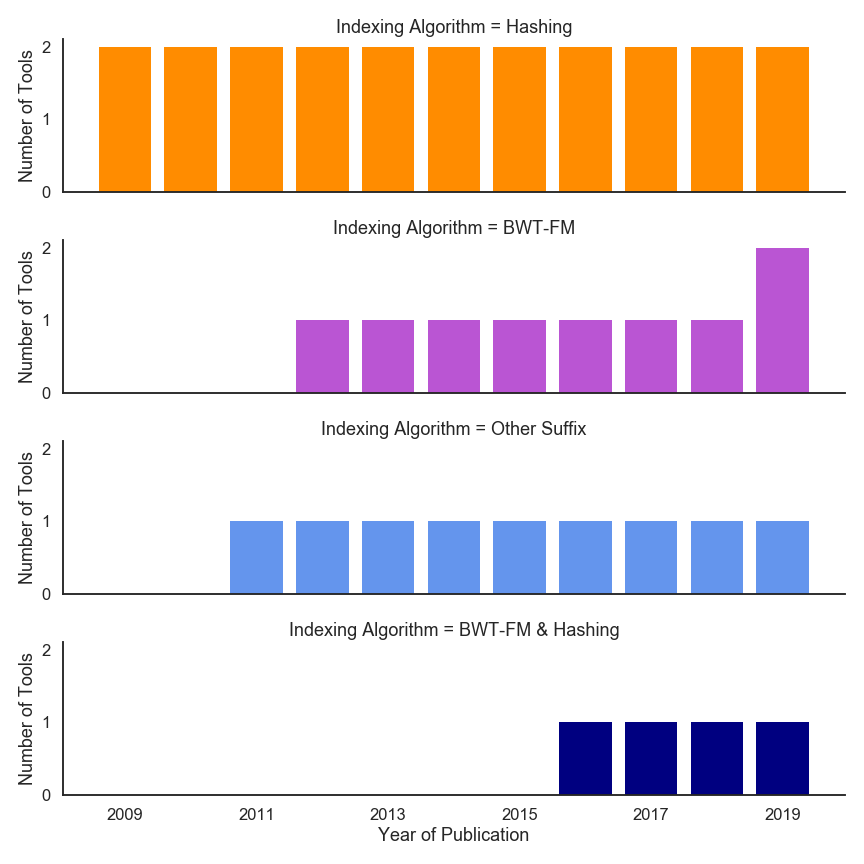


**Supplementary Figure 11. Histogram showing the cumulation of surveyed BS-Seq tools over time separated by the algorithm used for genome indexing.** Only stand alone BS-Seq aligners tools are included (not the wrappers of existing DNA-Seq aligners).

# **Supplementary Note 1**

We evaluate the effect of indexing on the end-to-end execution time of today’s read alignment algorithms. We align a single read of length 100 bp to the human reference genome (hg38) using BWA-MEM (with -*a* parameter selected to report all mapping locations). Building the index for the human reference genome takes 3,476 seconds. The read alignment step using BWA-MEM takes only 3.4 seconds after building the index for the human reference genome. Now we want to perform brute-force read alignment for the same read sequence and the same reference genome that we use for the BWA-MEM experiment. We divide the human reference genome into about 3.3 billion sequences, each of which is 100 bp long. That is, the first sequence is the first 100 bp of the reference genome and the second sequence is the segment that starts from the second bp of the reference genome and ends at the 101th bp and so forth for the other sequences. We then use Edlib’s global alignment tool (DP-based pairwise alignment) to check the similarity of the read sequence with each of the 3.3 billion generated sequences. We observe that Edlib takes about 24,200 seconds to complete the brute-force read alignment approach. This means that the indexing technique (and probably other filtering heuristics) used in BWA-MEM saves the execution time of read alignment by at least 7,100X. If we include the time needed to build the index in the total time of read alignment, then BWA-MEM is only 7X faster than the brute-force read alignment approach. Note that indexing the reference genome is performed only once for each reference genome.

#

# **Supplementary Note 2**

In our review, we define read alignment as a three-step process, which includes indexing, global positioning, and pairwise alignment. In this case, pairwise alignment is considered to be performed between a read and a section of the reference determined by global positioning. Alternatively, the entire process can be viewed as local alignment with respect to the reference, and global alignment with respect to the read. In this formulation, the read is aligned end-to-end to the best substring in the reference and is expressed as semi-global alignment[^13^](https://paperpile.com/c/c9hsou/37BfM).

We have simplified pairwise alignment into overarching algorithm classifications like Smith-Waterman or Needleman-Wunsch, but tools that use dynamic programming can be classified into subcategories that are beyond the scope of this review. For example, read alignment algorithms can choose to be gapless (ignoring some variants), compute edit distance (the minimum number of edits needed to convert one string into the other), or use an affine gap penalty where variants are weighted differently based on their length. It is also worth noting that BWT-based tools do not use seeding in the traditional sense, and seed classification might be performed differently.

# **Supplementary Note 3**

We first built the index data, then ran the alignment procedure and extracted the data in bam format. Some tools do not provide the output in bam format, so in this case we used samtools toolkit to convert sam output to bam output.

To install samtools from conda: conda install -c bioconda samtools

1) Bowtie2

Build index:

bowtie2-build <reference_in> <index_basename>

*reference_fasta: fasta file of reference genome

*index_basename: write index data to files with this basename

Mapping WGS data:

bowtie2 -x <index_basename> -1 <r1_fastq> -2 <r2_fastq> | samtools view -bS - > output.bam

*r1_fastq, r2_fastq: fastq files of the paired end reads

2) Bowtie

Build index:

bowtie-build <reference_in> <index_basename>

*reference_in: fasta file of reference genome

*index_basename: write index data to files with this basename

Mapping WGS data:

bowtie -S <index_basename> -1 <r1_fastq> -2 <r2_fastq> | samtools view -bS - > output.bam

3) BWA

Build index:

bwa index <reference_fasta>

Mapping WGS data:

bwa mem <reference_fasta> <r1_fastq> <r2_fastq> | samtools view -bS - > output.bam

4) GSNAP

Build index:

gmap_build -D <destination_directory_path> -d <genome_name> <reference_fasta>

Mapping WGS data:

gsnap -D <destination_directory_path> -d <genome_name> <r1_fastq> <r2_fastq> -A sam | samtools view -bS - > output.bam

5) HISAT2

Build index:

hisat2-build <reference_fasta> <index_basename>

Mapping WGS data:

hisat2 -q -x <index_basename> -1 <r1_fastq> -2 <r2_fastq> | samtools view -bS - > output.bam

*-q: input as fastq file

6) LAST

Build index:

lastdb -uNEAR -R01 <index_basename> <reference_fasta>

*-uNEAR and -R01 optional

Mapping WGS data:

lastal -Q1 <index_basename> <r1_fastq> <r2_fastq> | last-split > output.maf

*Q1: fastq-sanger format

7) minimap2

Build index:

Minimap2 -d <index_file> <reference_fasta>

* index file with “.mmi” extension

Mapping WGS data:

Minimap2 -a <index_file> <r1_fastq> | samtools view -bS - > output.bam

8) RMAP

rmap <read_fastq> -c <reference_fasta> -o output.sam | samtools view -bS - > output.bam

9) SMALT

Build index:

smalt index [options] <index_name> <reference_fasta>

Mapping WGS data:

smalt map <index_name> <r1_fastq> <r2_fastq> | samtools view -bS - > output.bam

10) SNAP

Build index:

snap-aligner <index_name> <reference_fasta> <index_dir_name>

Mapping WGS data:

snap-aligner paired <index_dir_name> <r1_fastq> <r2_fastq> -o output.bam

11) Subread

Build index:

subread-buildindex -o <index_name> <reference_fasta>

Mappins WGS data:

subread-align -t 1 -i <index_name> -r <r1_fastq> -R <r1_fastq> -o output.bam

#

# **Supplementary Note 4**

While a typical seed is a contiguous subsequence, a spaced seed contains in its sequence characters from a subsequence of the reference genome while ignoring the other characters of the same subsequence. Spaced seeds increase alignment sensitivity and enable hash tables to provide hits for both exact and inexact matches by ignoring certain bases of the seed. This approach was pioneered by PatternHunter[^4,14–16^](https://paperpile.com/c/c9hsou/tcuJc+8Eo4m+QtmFc+wuskL) in 2002 and has been adopted by 14 tools. A majority of the tools using spaced seeds are designed for short read technologies (Table 1). Spaced seeds can also be used in long read alignment to tolerate high error rates[^17^](https://paperpile.com/c/c9hsou/jx7aV). Another approach to account for the error rate of sequencing technologies involves generating seeds as prefixes of the read sequence. Generating the prefixes of the reads—as opposed to generating the suffixes—allows the read alignment algorithm to tolerate an increased error rate towards the end of a read[^18^](https://paperpile.com/c/c9hsou/Hjbpr). Other methods generate both suffix seeds and prefix seeds in order to tolerate large genetic variations[^19^](https://paperpile.com/c/c9hsou/iHpNx).

Instead of choosing a large number of seeds from each read, read alignment algorithms can choose only a small number of seeds that are apart from each other. This approach also allows larger genetic variations and sequencing errors that are located between every two adjacent seeds[^20^](https://paperpile.com/c/c9hsou/LuGxI). Most read alignment algorithms that follow this approach try to limit the number of differences that are located at the gaps in order to avoid aligning a read to highly dissimilar regions in the reference genome. This approach can be performed using seed extension followed by seed chaining. First, after finding a matching seed shared between a read and the reference genome, the read alignment algorithm extends the matching seed in both directions until there are no more exact matches (such extended seeds are called maximal exact matches (MEMs)^[21](https://paperpile.com/c/c9hsou/SiOxU)^). Second, the read alignment algorithm examines the gaps between every two adjacent extended seeds in the reference genome using a pairwise alignment algorithm[^22,23^](https://paperpile.com/c/c9hsou/iWbnK+QKTrH) to construct a longer chain of these adjacent extended seeds [^24^](https://paperpile.com/c/c9hsou/3gs93). The pairwise alignment can be performed end-to-end (e.g., global alignment) for two sequences of the same length[^22,23^](https://paperpile.com/c/c9hsou/iWbnK+QKTrH), or by using a local alignment algorithm[^11,25,26^](https://paperpile.com/c/c9hsou/967xO+N4xKg+H0tLp), where subsequences of the two given sequences are aligned. The two sequences can also be examined using a Hamming distance algorithm in cases where insertions or deletions are not allowed[^27^](https://paperpile.com/c/c9hsou/dBWbl). This seed chaining approach can also be applied to non-hashing-based read alignment algorithms, such as Bowtie2[^28^](https://paperpile.com/c/c9hsou/9ifJ1) and BWA-MEM[^29^](https://paperpile.com/c/c9hsou/3WJln). We observe that 54 read alignment algorithms out of the 107 surveyed alignment algorithms use a seed chaining approach.

**Supplementary Note 5**

Modern read alignment algorithms (e.g., Hobbes[^30^](https://paperpile.com/c/c9hsou/Bi9P8), Hobbes2[^31^](https://paperpile.com/c/c9hsou/FuwfU), Bitmapper[^32^](https://paperpile.com/c/c9hsou/Klk3Z), mrFAST[^33^](https://paperpile.com/c/c9hsou/tfiGB), RazerS[^34^](https://paperpile.com/c/c9hsou/SKPpm)) develop heuristics that quickly decide whether or not the computationally expensive DP calculation is needed—if not, significant time is saved by avoiding DP calculation. Such heuristics are called *pre-alignment filters*[*^35–39^*](https://paperpile.com/c/c9hsou/mVKx7+VfsUM+JYi3T+OQXc8+eTF5E), and they approximate the total number of differences between two sequences to determine if this count is greater than a threshold (Figure 1e). If so, these heuristics decide that the verification calculation is not needed due to high dissimilarity between the two sequences. Verification algorithms can also be accelerated using specialized or general-purpose hardware accelerators such as multi-core processors[^40–42^](https://paperpile.com/c/c9hsou/EgfUX+fPHdR+foeRi)^,[43](https://paperpile.com/c/c9hsou/WRV98)^.

# **Supplementary Note 6**

To obtain the nucleotide count in all bacterial genomes possessed by NCBI, we utilized the tool RepoPhlAn(<https://bitbucket.org/nsegata/repophlan>) to download via ftp.ncbi.nih.gov all genomes contained in the genomes/all subdirectory. Taxonomic identifiers were used to identify bacterial genomes and subsequently obtain a nucleotide count.

# obtain RepoPhlAn

wget <https://bitbucket.org/nsegata/repophlan/get/03f614c13cf0.zip>

unzip 03f614c13cf0.zip

cd 03f614c13cf0

# run RepoPhlAn

./run.sh # this can take upwards of 5 days to complete this step

cd out/microbes_<time_stamp>/fna

# count number of bacterial nucleotides

nohup ls -U | xargs -P 15 -I{} sh -c "bzcat {} | grep -v '>'| wc -m" | awk '{sum+=$1}END{print sum}' > ~/bacteria_bp_count.txt

# 676153484835

The human genome build GRCh38 was obtained from NCBI via ftp and nucleotides counted in the following way:

# download the human genome

wget -r <https://ftp.ncbi.nih.gov/genomes/Homo_sapiens/Assembled_chromosomes/seq/>*

# select just the fasta files

cd ftp.ncbi.nih.gov/genomes/Homo_sapiens/Assembled_chromosomes/seq/

ls | grep -v”\.fa\.” | xargs -I{} rm {}

#Uncompress

ls | xargs -I{} gunzip {}

# count nucleotides

ls *.fa | xargs -I{} sh -c " grep -v '>' {} | wc -m" | awk '{sum+=$1}END{print sum}' > ~/human_bp_count.txt

#3303852965

# compare the two

echo "`cat ~/bacteria_bp_count.txt` / `cat ~/human_bp_count.txt`" | bc -l

204.65604613702898246260

#

# **Supplementary Materials**

**Install the read alignment tools**

We have selected tools available on bioconda and have installed them using the following commands (Table S1).

**Public Sequence Data**

We used 10 WGS datasets for comparing the tools listed in Table S1. The SRA run accession numbers of the 10 datasets are as follows: ERR009309, ERR013127, ERR013138, ERR045708, ERR050158, ERR162843, ERR181410, ERR183377, SRR061640, and SRR360549. To download this data, we used the SRA toolkit which is available as a conda package.

Here are the commands that we used for the downloading process:

- To download sra toolkit: *conda install -c bioconda sra-tools*
- To download fastq files:
  - For single end fastq files: *fastq-dump <SRA_id>*
  - For paired end fastq files: *fastq-dump --split-files <SRA_id>*

**Compare the performance of the read alignments**

We recorded CPU time and RAM usage to compare the read alignment tools.

Tools were run in the UCLA’s Shared Hoffman2 Cluster.

Command that we used to submit our jobs in the cluster:

qsub -o <logfiles/> -e <logfiles/> -m bea -cwd -V -N <name_job> -l h_data=32G,highp,time=24:00:00 <exe_script>

*-m bea: define mailing rules

- b- start time of the job
- e- end time of the job
- a- time when the job is aborted

-cwd: changes the directory to where your executed file is, all log output will be created in this file unless you specify another directory (see command above output logs and error logs are directed to a folder named logfiles)

-V: export environment variables

-N: give a name to the submitted job

-l h_date: resource allocation

-l highp: submission of high priority jobs

-l time: job running time

**Statistical analyses**

We model expected CPU time *c_ij_* across all algorithms *i* and datasets *j* using the following gamma generalized linear mixed model regression

*log(E(c_ij_)) = α + a_j_ + β_1_ x Chain_of_seeds_ij_ + β_2_ x Indexing_ij_ + β_3_ x Year_of_publication_ij_ +*

*β’_4_ x Pairwise_alignment_ij_* (1)

where *α* is the intercept and *a_j_ ~ N(0,σ_j_)* is a data-level random intercept modelling the shared noise within each data set. *β_1_* is the effect of the *Chain_of_seeds* where *Chain_of_seeds* is coded as zero for no and one for yes. *β_2_* is the effect of Indexing, where *Indexing* is coded as 0 for BWT-FM and 1 for hashing or suffix array, depending on the group being compared to BWT-FM. *β_3_* is the effect of *Year_of_publication*, coded as a continuous variable of year scaled to have mean zero and variance one. *β_4_* is a vector with the effects of *Pairwise_alignment*, where *Pairwise_alignment* is a matrix of indicator variables for HD, Non-DP Heuristic, and SW algorithms, making NW the reference category. Parameter estimates are provided in (Table S3). We use a likelihood ratio test to test the effect of each variable discussed, e.g. year of publication or indexing, on the CPU time.

We use a similar model for the median across all datasets of the expected RAM usage med_*mem_i_*, i.e.

*log(E(*med_*mem_i_)) = α + β_1_ x Chain_of_seeds_ij_ + β_2_ x Indexing_ij_ + β_3_ x Year_of_publication_ij_ +*

*β_4_ x Pairwise_alignment_ij_* (2)

Parameter estimates are provided in (Table S4). Note that, as memory usage does not vary considerably within algorithms across data sets, we use the median expected RAM usage across all datasets for each algorithm.

**References**

1. [Langmead, B. & Salzberg, S. L. Fast gapped-read alignment with Bowtie 2. *Nature Methods* vol. 9 357–359 (2012).](http://paperpile.com/b/c9hsou/62YND)

2. [Langmead, B., Trapnell, C., Pop, M. & Salzberg, S. L. Ultrafast and memory-efficient alignment of short DNA sequences to the human genome. *Genome Biol.* **10**, R25 (2009).](http://paperpile.com/b/c9hsou/QwjKW)

3. [Li, H. & Durbin, R. Fast and accurate long-read alignment with Burrows–Wheeler transform. *Bioinformatics* **26**, 589–595 (2010).](http://paperpile.com/b/c9hsou/EfcuR)

4. [Wu, T. D. & Nacu, S. Fast and SNP-tolerant detection of complex variants and splicing in short reads. *Bioinformatics* **26**, 873–881 (2010).](http://paperpile.com/b/c9hsou/wuskL)

5. [Kim, D., Paggi, J. M., Park, C., Bennett, C. & Salzberg, S. L. Graph-based genome alignment and genotyping with HISAT2 and HISAT-genotype. *Nature Biotechnology* vol. 37 907–915 (2019).](http://paperpile.com/b/c9hsou/0y1S)

6. [Kiełbasa, S. M., Wan, R., Sato, K., Horton, P. & Frith, M. C. Adaptive seeds tame genomic sequence comparison. *Genome Res.* **21**, 487–493 (2011).](http://paperpile.com/b/c9hsou/NVhup)

7. [Li, H. Minimap2: pairwise alignment for nucleotide sequences. *Bioinformatics* **34**, 3094–3100 (2018).](http://paperpile.com/b/c9hsou/pylIM)

8. [Kim, D. *et al.* TopHat2: accurate alignment of transcriptomes in the presence of insertions, deletions and gene fusions. *Genome Biol.* **14**, R36 (2013).](http://paperpile.com/b/c9hsou/Aad1r)

9. [Hannes Ponsting, Z. N. SMALT - A New Mapper for DNA Sequencing Reads. (2010).](http://paperpile.com/b/c9hsou/BKuwz)

10. [Zaharia, M. *et al.* Faster and More Accurate Sequence Alignment with SNAP. *arXiv [cs.DS]* (2011).](http://paperpile.com/b/c9hsou/ZyN37)

11. [Liao, Y., Smyth, G. K. & Shi, W. The Subread aligner: fast, accurate and scalable read mapping by seed-and-vote. *Nucleic Acids Res.* **41**, e108 (2013).](http://paperpile.com/b/c9hsou/H0tLp)

12. [Artyomenko, A. *et al.* Long Single-Molecule Reads Can Resolve the Complexity of the Influenza Virus Composed of Rare, Closely Related Mutant Variants. *J. Comput. Biol.* **24**, 558–570 (2017).](http://paperpile.com/b/c9hsou/3upY4)

13. [Brudno, M. *et al.* Glocal alignment: finding rearrangements during alignment. *Bioinformatics* **19 Suppl 1**, i54–62 (2003).](http://paperpile.com/b/c9hsou/37BfM)

14. [Egidi, L. & Manzini, G. Better spaced seeds using Quadratic Residues. *Journal of Computer and System Sciences* vol. 79 1144–1155 (2013).](http://paperpile.com/b/c9hsou/tcuJc)

15. [Rizk, G. & Lavenier, D. GASSST: global alignment short sequence search tool. *Bioinformatics* **26**, 2534–2540 (2010).](http://paperpile.com/b/c9hsou/8Eo4m)

16. [Ma, B., Tromp, J. & Li, M. PatternHunter: faster and more sensitive homology search. *Bioinformatics* **18**, 440–445 (2002).](http://paperpile.com/b/c9hsou/QtmFc)

17. [Sović, I. *et al.* Fast and sensitive mapping of nanopore sequencing reads with GraphMap. *Nat. Commun.* **7**, 11307 (2016).](http://paperpile.com/b/c9hsou/jx7aV)

18. [Kircher, M., Heyn, P. & Kelso, J. Addressing challenges in the production and analysis of illumina sequencing data. *BMC Genomics* **12**, 382 (2011).](http://paperpile.com/b/c9hsou/Hjbpr)

19. [Emde, A.-K. *et al.* Detecting genomic indel variants with exact breakpoints in single- and paired-end sequencing data using SplazerS. *Bioinformatics* **28**, 619–627 (2012).](http://paperpile.com/b/c9hsou/iHpNx)

20. [Kloosterman, W. P. *et al.* Characteristics of de novo structural changes in the human genome. *Genome Res.* **25**, 792–801 (2015).](http://paperpile.com/b/c9hsou/LuGxI)

21. [Delcher, A. L., Phillippy, A., Carlton, J. & Salzberg, S. L. Fast algorithms for large-scale genome alignment and comparison. *Nucleic Acids Res.* **30**, 2478–2483 (2002).](http://paperpile.com/b/c9hsou/SiOxU)

22. [Slater, G. S. C. & Birney, E. Automated generation of heuristics for biological sequence comparison. *BMC Bioinformatics* **6**, 31 (2005).](http://paperpile.com/b/c9hsou/iWbnK)

23. [Siragusa, E., Weese, D. & Reinert, K. Fast and accurate read mapping with approximate seeds and multiple backtracking. *Nucleic Acids Res.* **41**, e78 (2013).](http://paperpile.com/b/c9hsou/QKTrH)

24. [Mäkinen, V. & Sahlin, K. Chaining with overlaps revisited. (2020).](http://paperpile.com/b/c9hsou/3gs93)

25. [Chen, S., Wang, A. & Li, L. M. SEME: A Fast Mapper of Illumina Sequencing Reads with Statistical Evaluation. *Lecture Notes in Computer Science* 14–29 (2013) doi:](http://paperpile.com/b/c9hsou/967xO)[10.1007/978-3-642-37195-0_2](http://dx.doi.org/10.1007/978-3-642-37195-0_2)[.](http://paperpile.com/b/c9hsou/967xO)

26. [David, M., Dzamba, M., Lister, D., Ilie, L. & Brudno, M. SHRiMP2: sensitive yet practical SHort Read Mapping. *Bioinformatics* **27**, 1011–1012 (2011).](http://paperpile.com/b/c9hsou/N4xKg)

27. [Hach, F. *et al.* mrsFAST: a cache-oblivious algorithm for short-read mapping. *Nat. Methods* **7**, 576–577 (2010).](http://paperpile.com/b/c9hsou/dBWbl)

28. [Langmead, B. & Salzberg, S. L. Fast gapped-read alignment with Bowtie 2. *Nat. Methods* **9**, 357–359 (2012).](http://paperpile.com/b/c9hsou/9ifJ1)

29. [Li, H. Aligning sequence reads, clone sequences and assembly contigs with BWA-MEM. *arXiv [q-bio.GN]* (2013).](http://paperpile.com/b/c9hsou/3WJln)

30. [Ahmadi, A. *et al.* Hobbes: optimized gram-based methods for efficient read alignment. *Nucleic Acids Res.* **40**, e41 (2012).](http://paperpile.com/b/c9hsou/Bi9P8)

31. [Kim, J., Li, C. & Xie, X. Improving read mapping using additional prefix grams. *BMC Bioinformatics* **15**, 42 (2014).](http://paperpile.com/b/c9hsou/FuwfU)

32. [Cheng, H., Jiang, H., Yang, J., Xu, Y. & Shang, Y. BitMapper: an efficient all-mapper based on bit-vector computing. *BMC Bioinformatics* **16**, 192 (2015).](http://paperpile.com/b/c9hsou/Klk3Z)

33. [Alkan, C. *et al.* Personalized copy number and segmental duplication maps using next-generation sequencing. *Nat. Genet.* **41**, 1061–1067 (2009).](http://paperpile.com/b/c9hsou/tfiGB)

34. [Weese, D., Emde, A.-K., Rausch, T., Döring, A. & Reinert, K. RazerS--fast read mapping with sensitivity control. *Genome Res.* **19**, 1646–1654 (2009).](http://paperpile.com/b/c9hsou/SKPpm)

35. [Alser, M., Hassan, H., Kumar, A., Mutlu, O. & Alkan, C. Shouji: A Fast and Efficient Pre-Alignment Filter for Sequence Alignment. *Bioinformatics* (2019) doi:](http://paperpile.com/b/c9hsou/mVKx7)[10.1093/bioinformatics/btz234](http://dx.doi.org/10.1093/bioinformatics/btz234)[.](http://paperpile.com/b/c9hsou/mVKx7)

36. [Alser, M. *et al.* GateKeeper: a new hardware architecture for accelerating pre-alignment in DNA short read mapping. *Bioinformatics* **33**, 3355–3363 (2017).](http://paperpile.com/b/c9hsou/VfsUM)

37. [Alser, M., Mutlu, O. & Alkan, C. MAGNET: Understanding and Improving the Accuracy of Genome Pre-Alignment Filtering. *arXiv [q-bio.GN]* (2017).](http://paperpile.com/b/c9hsou/JYi3T)

38. [Kim, J. S. *et al.* GRIM-Filter: Fast seed location filtering in DNA read mapping using processing-in-memory technologies. *BMC Genomics* **19**, 89 (2018).](http://paperpile.com/b/c9hsou/OQXc8)

39. [Alser, M., Shahroodi, T., Gómez-Luna, J., Alkan, C. & Mutlu, O. SneakySnake: A Fast and Accurate Universal Genome Pre-Alignment Filter for CPUs, GPUs, and FPGAs. *Bioinformatics* (2020) doi:](http://paperpile.com/b/c9hsou/eTF5E)[10.1093/bioinformatics/btaa1015](http://dx.doi.org/10.1093/bioinformatics/btaa1015)[.](http://paperpile.com/b/c9hsou/eTF5E)

40. [Zhang, J. *et al.* BGSA: A Bit-Parallel Global Sequence Alignment Toolkit for Multi-core and Many-core Architectures. *Bioinformatics* (2018) doi:](http://paperpile.com/b/c9hsou/EgfUX)[10.1093/bioinformatics/bty930](http://dx.doi.org/10.1093/bioinformatics/bty930)[.](http://paperpile.com/b/c9hsou/EgfUX)

41. [Turakhia, Y., Goenka, S. D., Bejerano, G. & Dally, W. J. Darwin-WGA: A Co-processor Provides Increased Sensitivity in Whole Genome Alignments with High Speedup. *2019 IEEE International Symposium on High Performance Computer Architecture (HPCA)* (2019) doi:](http://paperpile.com/b/c9hsou/fPHdR)[10.1109/hpca.2019.00050](http://dx.doi.org/10.1109/hpca.2019.00050)[.](http://paperpile.com/b/c9hsou/fPHdR)

42. [Cali, D. S. *et al.* GenASM: A High-Performance, Low-Power Approximate String Matching Acceleration Framework for Genome Sequence Analysis. in *2020 53rd Annual IEEE/ACM International Symposium on Microarchitecture (MICRO)* 951–966 (2020).](http://paperpile.com/b/c9hsou/foeRi)

43. [Alser, M. *et al.* Accelerating Genome Analysis: A Primer on an Ongoing Journey. *IEEE Micro* vol. 40 65–75 (2020).](http://paperpile.com/b/c9hsou/WRV98)
