# Supplementary material for: Technology dictates algorithms: recent developments in read alignment
Source: Genome Biol. 2021 Aug 26;22:249. doi: 10.1186/s13059-021-02443-7 (PMC8390189; doi:10.1186/s13059-021-02443-7)
Supplement: Supplementary file 2 — Additional file 2. Review history. [file 13059_2021_2443_MOESM2_ESM.docx]

**1^st^ round**

**Reviewer 1**

**Is the topic of the article timely and of interest to a wide range of readers?**

Yes

**Is the article well written and presented in a logical way?**

Yes

**Do the authors cover the relevant literature in an accurate and balanced way?**

Yes

**Do the authors provide a useful synthesis of the topic?**

Yes

**Do the authors provide insightful discussion of the future directions for the field?**

Yes: Sequence alignment is the fundamental step for a myriad of downstream applications in genomics. Although this has been a subject of research for over multiple decades, given the rapid growth of new sequencing technologies and applications, a detailed understanding of the use case for a specific aligner should be clear to the community. There are many open source aligners that are available at one’s disposal for analyzing a specific dataset. Authors of this manuscript laid out specific building blocks for the existing aligners and performed a rigorous analysis for the time and memory performance. Subsequently they did statistical modeling to assess the contribution of a particular attribute that goes into the final performance of the aligner. As I outlined in my review, there is scope of improvement in the manuscript to make it acceptable. But in my opinion the authors did a great job in setting up a principle way for understanding and building future aligners.

**Do any tables, figures or boxes that are present enhance the manuscript?**

Yes

**Would the manuscript benefit from any additional tables, figures or boxes?**

Yes

**Comments to author**

The manuscript "Technology dictates algorithms: Recent developments in read alignment" by Alser et al. presents a rigorous description of the ever-growing field of "biological sequence alignment" that serves as a core engine for multitudes of downstream applications such as gene expression quantification, differential expression, etc. Alser et al. surveyed 107 different alignment algorithms developed in the span of 32 years from 1988 to 2020. The manuscript rightfully captured various heterogeneity of biological sequences, from protocol type to length of the reads to organism-specific challenges.

Given the astronomic growth in the publicly available sequence datasets and the alignment algorithms, the manuscript is of significant importance to analyze and dissect what algorithm is best suited for a specific purpose. Additionally, the manuscript provides a historical perspective that is crucial in understanding the evolution of the alignment algorithms. I want to particularly commend the authors' efforts to reproduce the plots and tables via the open-source GitHub repository.

While the paper nicely ties together the subject matter, I have the following concerns about the present manuscript,

-Major Concerns-

The manuscript quite rightly outlined the challenges of a modern-day aligner concerning the type of sequences it is designed to tackle. It describes that "Error rate", "Genome Coverage", "Global position" and "Local pairwise alignment" etc., play a crucial role in determining the usefulness of an aligner. It seems the real challenge is to find out the right middle ground between specificity: keeping a set of heuristics to accommodate possible approximate matches, sensitivity: being too conservative about accepting an alignment. Leaning towards either direction can lead to undesirable outcomes. Therefore the algorithm has to be tailor-made for the specific application.

My primary concern with the manuscript is that it does not provide an in-depth analysis of how these different attributes affect the accuracy of the aligners. While the rigorous time and memory benchmark are crucial for choosing one method over the other, the fundamental element of choice lies in the alignment's actual accuracy. To be specific, a user selects an aligner by judging it on the scale of specificity and sensitivity.

The manuscript bases the results on a range of real data samples, reflecting the performance across a heterogeneous set of datasets. For benchmarking the accuracy of these tools, I would highly recommend using simulated read datasets incorporating different parameters outlined in the manuscript. Varying "Error rate", "Genome Coverage", "Global position", "Local pairwise alignment", "Genomic variants", "Read length" as tunable parameters, one could single out the effect of each of these attributes for a particular alignment algorithm. Available DNA-seq/RNA-seq simulators (e.g., ART or more recent https://github.com/schmeing/ReSeq) for single-end, paired-end reads sort reads could be used for benchmarking DNA-seq specific aligners. Additionally, PacBio long-read simulators (such as https://github.com/rrwick/Badread) can be used to generate long reads with realistic error profiles. It is not necessary, but the authors can also perform a similar analysis or point to an already existing benchmark for metagenomic analysis. A realistic measure of accuracy in the alignment is quite straightforward, as it depends on the reads that are correctly mapped back to the location of origin, determining true positive/ false positives, etc. Although it is understandable under a high variation rate, it might be infeasible to map a read to a unique location correctly, but those cases will be infrequent.

Some similar classification is done for time and memory usage can also be implemented for the accuracy metric. All the design principles that are discussed in the manuscripts, such as hashing to BWT-FM based indexing or using a mixed alignment scheme to form the final set of alignment, can have a recognizable effect on accuracy.

In my opinion, such a measure will increase the usefulness of the manuscript to a great extent.

-Other concerns-

1. The plots of figure 4 correspond to the performance of 11 different alignment methodologies on various experiments. I guess the multiple points with the same color for each method refer to the experiments. It would be helpful to mention the legend in the caption.

2. The corresponding paragraph referring to Figure 4(c) mentions that there is not much difference between the Hashing based methods and the BWT-FM based methods. It seems SMALT is a hashing based method that stands out from other hashing based methods in terms of run time, while SNAP is the hashing based method that takes the highest memory. It will be interesting to discuss the reason for such a performance difference.

3. The colors used in Figure 4 are not very distinctive to me. I had a hard time identifying the tools back, especially when the colors are too similar. Given that there are multiple experiments for one tool, it would help identify a tool by an arrow whenever possible (specifically for figure 4f).

For example, the text in the manuscript mentions that "Despite the overall longer runtime of Hamming-distance-based methods, the latest hashing based tool hashing based too (e.g., HISAT2) provide a comparable..", unfortunately, I could not identify HISAT2 back from the diagram. Using a different legend might be useful.

4. Table 2 classifies the alignment algorithms from a practical standpoint based on metrics such as "ease of implementation" etc. I am curious how these metrics are computed.

5. "; the shared prefix between the read and the genome is stored only once and used by all the reads with that prefix." - I could not comprehend this sentence correctly; the suffix tree should be independent of the reads; it's part of the index made on the reference sequence. An explanation would be beneficial.

6. "Unlike a hash table, a suffix tree allows searching for both the exact and inexact match seeds by walking through.."

The initial seeding strategy can have a variable length of k-mers for selecting prefixes from the suffix array. Still, I could not understand how the suffix tree built on the index can allow inexact search if an additional set of heuristics is not employed.

7. \beta is used as a coefficient for the generalized linear mixed model regression. The values themselves do not express a lot, in my opinion (except their signs). It would be really helpful if some explanation can be added with positive and negative values for the corresponding coefficient.

8. Page 23 paragraph starting from line 29 describes the standard process for transcriptome quantification using DNA-seq aligners. The following paragraph briefly describes the use of light-weight mappers to do transcript level read assignment. There are some issues with the generalization in the description. The commonly used pseudo-alignment-based methods, such as Kallisto, uses De-Bruijn graphs. In contrast, the other tool, Salmon, uses k-mer hash in conjunction with a Suffix Array index (using mapper such as RapMap) with additional heuristics to speed up the mapping process.

Moreover, Salmon can provide mapping locations and a mapping score. However, as the authors rightly pointed out, none of these tools provide CIGAR strings or any other alignment profiles for further analysis. I would suggest the authors to rewrite the sections with a more detailed treatment of the individual methods given both of the tools are highly cited and used.

9. Referring to page 15 line 4: it will be helpful to elaborate on the kind of heuristics that the aligner algorithms used, such as bidirectional jump, skipping, hamming distance thresholding, etc. As with other aspects of the alignment pipeline, the seed extension heuristic can have a profound effect on the alignment results.

**Reviewer 2**

**Is the topic of the article timely and of interest to a wide range of readers?**

Yes

**Is the article well written and presented in a logical way?**

Yes

**Do the authors cover the relevant literature in an accurate and balanced way?**

Yes

**Do the authors provide a useful synthesis of the topic?**

Yes

**Do the authors provide insightful discussion of the future directions for the field?**

Yes: The authors indeed provided insightful discussion of the future directions for the field.**Do any tables, figures or boxes that are present enhance the manuscript?**

Yes

**Would the manuscript benefit from any additional tables, figures or boxes?**

Yes

**Comments to author**

This is a very-well written review paper with broad vision and unique perspective. The algorithmic details of the alignments methods were clearly explained to a proper depth, and all methods are well categorized based on the underlying algorithms used. The advantages and limitations of each category are convincingly described and well illustrated with tables and figures. I also like the discussion about the challenges and opportunities for reads alignments with the recent advancement of long-reads technologies

Two suggestions:

1, 10 WGS samples were used to evaluate the chosen methods. I believe they are all short-reads data. If so, it would be more helpful if some long-reads data can be included to evaluate the mentioned long-reads aligners.

2, In the GitHub repo

(https://github.com/Mangul-Lab-USC/review_technology_dictates_algorithms) users were directed to the repository's wiki for more information. But I didn't find the wiki page. A detailed description on how to use the provided scripts to reproduce the experimental results in the manuscript would be very helpful to the community.

**Authors’ response**

**Reviewer 1**

The manuscript quite rightly outlined the challenges of a modern-day aligner concerning the type of sequences it is designed to tackle. It describes that "Error rate", "Genome Coverage", "Global position" and "Local pairwise alignment" etc., play a crucial role in determining the usefulness of an aligner. It seems the real challenge is to find out the right middle ground between specificity: keeping a set of heuristics to accommodate possible approximate matches, sensitivity: being too conservative about accepting an alignment. Leaning towards either direction can lead to undesirable outcomes. Therefore the algorithm has to be tailor-made for the specific application.

My primary concern with the manuscript is that it does not provide an in-depth analysis of how these different attributes affect the accuracy of the aligners. While the rigorous time and memory benchmark are crucial for choosing one method over the other, the fundamental element of choice lies in the alignment's actual accuracy. To be specific, a user selects an aligner by judging it on the scale of specificity and sensitivity. The manuscript bases the results on a range of real data samples, reflecting the performance across a heterogeneous set of datasets. For benchmarking the accuracy of these tools, I would highly recommend using simulated read datasets incorporating different parameters outlined in the manuscript. Varying "Error rate", "Genome Coverage", "Global position", "Local pairwise alignment", "Genomic variants", "Read length" as tunable parameters, one could single out the effect of each of these attributes for a particular alignment algorithm.

Available DNA-seq/RNA-seq simulators (e.g., ART or more recent

https://urldefense.com/v3/__https://github.com/schmeing/ReSeq__;!!LIr3w8kk_Xxm!6t79

Yi8uYPnGb6lb0G_SMZ1pLX-jpHO-ikoh20FtEGuRbw39nh20n3A68d_Q5g$ ) for single-end, paired-end reads sort reads could be used for benchmarking DNA-seq specific aligners.

Additionally, PacBio long-read simulators (such as

https://urldefense.com/v3/__https://github.com/rrwick/Badread__;!!LIr3w8kk_Xxm!6t79Y

i8uYPnGb6lb0G_SMZ1pLX-jpHO-ikoh20FtEGuRbw39nh20n3Dgt6WhPg$ ) can be used to generate long reads with realistic error profiles. It is not necessary, but the authors can also perform a similar analysis or point to an already existing benchmark for metagenomic analysis. A realistic measure of accuracy in the alignment is quite straightforward, as it depends on the reads that are correctly mapped back to the location of origin, determining true positive/ false positives, etc. Although it is understandable under a high variation rate, it might be infeasible to map a read to a unique location correctly, but those cases will be infrequent. Some similar classification is done for time and memory usage can also be implemented for the accuracy metric. All the design principles that are discussed in the manuscripts, such as hashing to BWT-FM based indexing or using a mixed alignment scheme to form the final set of alignment, can have a recognizable effect on accuracy. In my opinion, such a measure will increase the usefulness of the manuscript to a great extent.

- We thank the reviewer for this valuable feedback. We agree on the importance of benchmarking the accuracy of different read alignment algorithms (and metagenomic analysis algorithms). However, as we are aiming to write a comprehensive review paper instead of a benchmarking paper, we believe this feedback is out of the scope of our manuscript. We prefer to keep the paper as a review paper that targets a broader audience and lists the historical perspective of algorithmic foundations and methodologies behind read alignment. We believe evaluating the accuracy using different metrics and datasets is still out of the scope of our review paper. The feedback provided by the reviewer, the suggested read simulator and suggested performance metrics are all very helpful and we will consider them in our another ongoing study that aims to evaluate the robustness and reproducibility of existing read alignment algorithms.

The plots of figure 4 correspond to the performance of 11 different alignment methodologies on various experiments. I guess the multiple points with the same color for each method refer to the experiments. It would be helpful to mention the legend in the caption.

- We mention the legend in the caption of Figure 4, as we show below:

The corresponding paragraph referring to Figure 4(c) mentions that there is not much difference between the Hashing based methods and the BWT-FM based methods. It seems SMALT is a hashing based method that stands out from other hashing based methods in terms of run time, while SNAP is the hashing based method that takes the highest memory. It will be interesting to discuss the reason for such a performance difference.

- We thank the reviewer for the feedback. We address this comment by clarifying two outliers to the observed performance. We added two sentences to explain the reasons for the high execution time of SMALT and the high memory footprint of SNAP, as we show below:

The colors used in Figure 4 are not very distinctive to me. I had a hard time identifying the tools back, especially when the colors are too similar. Given that there are multiple experiments for one tool, it would help identify a tool by an arrow whenever possible (specifically for figure 4f).

For example, the text in the manuscript mentions that "Despite the overall longer runtime of Hamming-distance-based methods, the latest hashing based tool hashing based too (e.g., HISAT2) provide a comparable..", unfortunately, I could not identify HISAT2 back from the diagram. Using a different legend might be useful.

- We thank the reviewer for this helpful feedback. To address the reviewer’s comment and easily distinguish each aligner performance, we changed four figures, Fig 4, Supplementary Fig 1, Supplementary Fig 2, and Supplementary Fig 4, by 1) adding another label to their x-axises to indicate the corresponding aligner tool and 2) vertically grouping all data points together (by enabling dodge option in Seaborn) that belong to each aligner tool, as we provide below:

Table 2 classifies the alignment algorithms from a practical standpoint based on metrics such as "ease of implementation" etc. I am curious how these metrics are computed.

- We define the “ease of implementation” as the ability to quickly implement such a read alignment algorithm and its indexing technique, flexibly apply some changes to it, and easily understand its working principle.

- To address this feedback, we added a new sentence to the caption of Table 2, as we show below:

"; the shared prefix between the read and the genome is stored only once and used by all the reads with that prefix." - I could not comprehend this sentence correctly; the suffix tree should be independent of the reads; it's part of the index made on the reference sequence. An explanation would be beneficial.

- We agree with the reviewer on the unclarity of this sentence. To address this issue, we change the sentence, as we provide below:

"Unlike a hash table, a suffix tree allows searching for both the exact and inexact match seeds by walking through.."

The initial seeding strategy can have a variable length of k-mers for selecting prefixes from the suffix array. Still, I could not understand how the suffix tree built on the index can allow inexact search if an additional set of heuristics is not employed.

- There are several algorithms that can perform inexact matching directly on suffix arrays, suffix trie, and other structures. We refer the reviewer to the following two papers as examples of such algorithms.

- Ghodsi, Mohammadreza, and Mihai Pop. "Inexact local alignment search over suffix arrays." 2009 IEEE international conference on bioinformatics and biomedicine. IEEE, 2009.

- Ukkonen, Esko. "Approximate string-matching over suffix trees." Annual Symposium on Combinatorial Pattern Matching. Springer, Berlin, Heidelberg, 1993.

It can be briefly done by traversing several branches based on the shared prefix between these branches, where each detour from one branch to another can allow for inexact matching at the corresponding location of the detour.

- To address this issue and make it more clear, we added the two references to the mentioned sentence in the paper, as we provide below:

\beta is used as a coefficient for the generalized linear mixed model regression. The values themselves do not express a lot, in my opinion (except their signs). It would be really helpful if some explanation can be added with positive and negative values for the corresponding coefficient.

- Thanks for the valuable comments. We agree with the reviewer’s comment. To address this issue, we replace all occurrences of \beta in the paper with the corresponding amount of increase/decrease, as we provide below:

Page 23 paragraph starting from line 29 describes the standard process for transcriptome quantification using DNA-seq aligners. The following paragraph briefly describes the use of light-weight mappers to do transcript level read assignment. There are some issues with the generalization in the description. The commonly used pseudo-alignment-based methods, such as Kallisto, uses De-Bruijn graphs. In contrast, the other tool, Salmon, uses k-mer hash in conjunction with a Suffix Array index (using mapper such as RapMap) with additional heuristics to speed up the mapping process.

Moreover, Salmon can provide mapping locations and a mapping score. However, as the authors rightly pointed out, none of these tools provide CIGAR strings or any other alignment profiles for further analysis. I would suggest the authors to rewrite the sections with a more detailed treatment of the individual methods given both of the tools are highly cited and used.

- We thank the reviewer for the feedback. We agree on the fundamental difference between Kallisto and Salmon. To address this issue and make the description more comprehensive, we added the following paragraphs to the paper, as we provide below:

Referring to page 15 line 4: it will be helpful to elaborate on the kind of heuristics that the aligner algorithms used, such as bidirectional jump, skipping, hamming distance thresholding, etc. As with other aspects of the alignment pipeline, the seed extension heuristic can have a profound effect on the alignment results.

- We thank the reviewer for suggesting to elaborate on the heuristics used in read alignment. To address this issue, we added a new paragraph to the “Supplementary Note 4” section and referenced it in the sentence that the reviewer highlighted, as we provide below. We also mentioned other heuristics used to accelerate pairwise sequence alignment in the “Supplementary Note 5” section in the submitted version.

**Reviewer 2**

10 WGS samples were used to evaluate the chosen methods. I believe they are all short-reads data. If so, it would be more helpful if some long-reads data can be included to evaluate the mentioned long-reads aligners.

- We thank the reviewer for this feedback. We agree on the importance of using long reads for the evaluation. However, we believe that using long reads will not provide additional insights about the algorithmic developments of read alignment for the following four reasons: 1) Our goal in this paper is to review the historical perspective of the read alignment algorithmic development and foundations and not benchmarking existing tool nor finding “best” read alignment tool. 2) As we mention in the “Influence of long-read technologies on the development of novel read alignment algorithm” section, most of the long read alignment algorithms are still following the three key steps that we observe in short read alignment algorithms. 3) In Table 1, we observe that only a few read alignment algorithms (LAMSA, Magic-BLAST, BLAST, NGMLR, lordFAST, minimap2, YAHA, SNAP, BWA-SW, GraphMap, GraphMap2, conLSH, rHAT, BLASR) support long reads (read length of >= 8000). We also observe that only two of these tools (SNAP and minimap2) are available to us on bioconda. Hence, we will not have enough tools on bioconda for our long read evaluation to compare using different performance metrics. 4) We already considered evaluating the performance of the read alignment algorithms that are proposed after 2013 (the first ONT sequencing machine was available in 2014), which can support longer read lengths (> 200 bp), in Figure 4e and Supplementary Figure 1.

In the GitHub repo

(https://urldefense.com/v3/__https://github.com/Mangul-Lab-USC/review_technology_dic

tates_algorithms__;!!LIr3w8kk_Xxm!6t79Yi8uYPnGb6lb0G_SMZ1pLX-jpHO-ikoh20FtEGuRb

w39nh20n3BRr8JZ2g$) users were directed to the repository's wiki for more information.

But I didn't find the wiki page. A detailed description on how to use the provided scripts to reproduce the experimental results in the manuscript would be very helpful to the community.

- We thank the reviewer for this important feedback on reproducibility and user-friendliness. To address this feedback, we improved the GitHub page of our paper to include more details about the project in general and on how to reproduce the same results we have in our paper, as we provide in: https://github.com/Mangul-Lab-USC/review-technology-dictates-algorithms
